# Proscillaridin A induces apoptosis and suppresses non-small-cell lung cancer tumor growth via calcium-induced DR4 upregulation

**DOI:** 10.1038/s41419-018-0733-4

**Published:** 2018-06-13

**Authors:** Run-Ze Li, Xing-Xing Fan, Fu-Gang Duan, Ze-Bo Jiang, Hu-Dan Pan, Lian-Xiang Luo, Yan-Ling Zhou, Ying Li, Ying-Jia Yao, Xiao-Jun Yao, Elaine Lai-Han Leung, Liang Liu

**Affiliations:** 1 State Key Laboratory of Quality Research in Chinese Medicine/Macau Institute for Applied Research in Medicine and Health, Macau University of Science and Technology, Macau (SAR), China; 2 Department of Thoracic Surgery, Guangzhou Institute of Respiratory Health and State Key Laboratory of Respiratory Disease, The First Affiliated Hospital of Guangzhou Medical University, Guangzhou, Guangdong, China; 3 Respiratory Medicine Department, Taihe Hospital, Hubei University of Medicine, Hubei, China

## Abstract

Non-small-cell lung cancer (NSCLC) is the predominant histological type of lung cancer and is characterized by the highest mortality and incidence rates among these types of malignancies. Cardiac glycosides, a class of natural products, have been identified as a potential type of chemotherapeutic agent. This study aims to investigate the anti-cancer effects and the mechanisms of action of Proscillaridin A (P.A) in NSCLC cells. In vitro sodium–potassium pump (Na^+^/K^+^ ATPase) enzyme assays indicated that P.A is a direct Na^+^/K^+^ ATPase inhibitor. P.A showed potent cytotoxic effects in NSCLC cells at nanomolar levels. Treatment mechanism studies indicated that P.A elevated Ca^2+^ levels, activated the AMPK pathway and downregulated phosphorylation of ACC and mTOR. Subsequently, P.A increased death receptor 4 (DR4) expression and downregulated NF–κB. Interestingly, P.A selectively suppressed EGFR activation in EGFR mutant cells but not in EGFR wild-type cells. In vivo, P.A significantly suppressed tumor growth in nude mice compared to vehicle-treated mice. Compared with the Afatinib treatment group, P.A displayed less pharmaceutical toxicity, as the body weight of mice treated with P.A did not decrease as much as those treated with Afatinib. Consistent changes in protein levels were obtained from western blotting analysis of tumors and cell lines. Immunohistochemistry analysis of the tumors from P.A-treated mice showed a significant suppression of EGFR phosphorylation (Tyr 1173) and reduction of the cell proliferation marker Ki-67. Taken together, our results suggest that P.A is a promising anti-cancer therapeutic candidate for NSCLC.

## Introduction

Cancer is one of the leading causes of death both in China and worldwide^1^. Lung cancer has the highest incidence and mortality rates among all malignancies^2^. Over 1.6 million cases of lung cancer are diagnosed each year, accounting for 13% of all new cancer diagnoses. Further, 1.4 million deaths per year are attributed to lung cancer, accounting for 18% of all cancer-related deaths^3^. Among the various types of lung cancers, non-small-cell lung cancer (NSCLC) comprises 80–85% of all cases^4^; unfortunately, more than 70% of these cases are diagnosed as unresectable, advanced stage tumors^5^. Although many medical intervention methods have been proposed, the prognosis for NSCLC patients remains poor, with an 18% 5-year overall survival (OS) rate across all stages^2^.

To date, the main lung cancer treatment strategy involves the direct inhibition of tumor cell growth by cytotoxic agents and targeted therapies^6^. However, drug resistance is common and treatments are limited, thus new strategies have been developed including those affecting intracellular calcium (Ca^2+^) homeostasis. Ca^2+^, a second messenger, is involved in various fundamental functions, such as the regulation of gene transcription and cellular metabolic activity, which affects both cell proliferation and cell death^7^. It has been demonstrated that Ca^2+^ levels could be altered in different tumor types—such as ovarian, prostate, brain, and breast cancer—by altering Ca^2+^ channels and disrupting pump activity through post-translational modification^8–10^. Thus, inducing cell death by increasing the intracellular Ca^2+^ levels may be a novel method for the treatment of cancer. Another important P-type ATPase family member that can affect Ca^2+^ concentration is the Na^+^/K^+^ ATPase^11^. Analyses have demonstrated altered expression levels of Na^+^/K^+^ ATPase subunits in lung cancer cells, specifically, overexpression of the α1 and α3 subunits^12,13^. Numerous investigations have shown that cardiac glycosides (inhibitors of Na^+^/K^+^ ATPase) could induce apoptosis in tumor cells^14–16^.

Traditional Chinese medicines (TCM) are a treasure trove of drugs that may be utilized for the treatment of different diseases. The clinical applications of Artemisinin in the treatment of malaria and Berberine in the treatment of type II diabetes aroused research interests regarding TCM^17^. In our previous studies, we screened a library of 800 natural compounds using MTT assays and identified Proscillaridin A (P.A) as having a relatively large anti-cancer effect in A549 and H1975 NSCLC cell lines^18^. In this study, we aimed to further investigate the mechanism of action of P.A, a constituent of squill-*Drimia maritima*, as a treatment for NSCLC. P.A has been previously investigated in several different kinds of cancer cells by other groups. It has been demonstrated that P.A can inhibit HIF-1α and reduce cell proliferation in prostate carcinoma and hepatocellular carcinoma^19^. Other researchers have demonstrated that the anti-cancer effect of P.A occurs through inhibition of DNA topoisomerase I and II in breast cancer^20,21^. P.A also induces apoptosis of cancer cells and suppresses tumor xenograft growth in a glioblastoma model^22^.

However, to our knowledge, there is currently no mechanistic study of P.A in NSCLC cells. Therefore, in this study, we aimed to first investigate the cytotoxicity of P.A in a panel of NSCLC cells by MTT assay. Second, we determined the functional effects of P.A by flow cytometry and colony formation assays. Then, the treatment mechanism of P.A was explored via sodium/potassium ATPase enzyme activity, flow cytometry, Western blotting and small interfering RNA transfection methods in NSCLC cells. Finally, we examined the in vivo efficacy of P.A in GFP-transformed H1975 stable cells in a xenograft mouse model.

## Results

### NSCLC cells are sensitive to P.A treatment

**Fig. 1:**
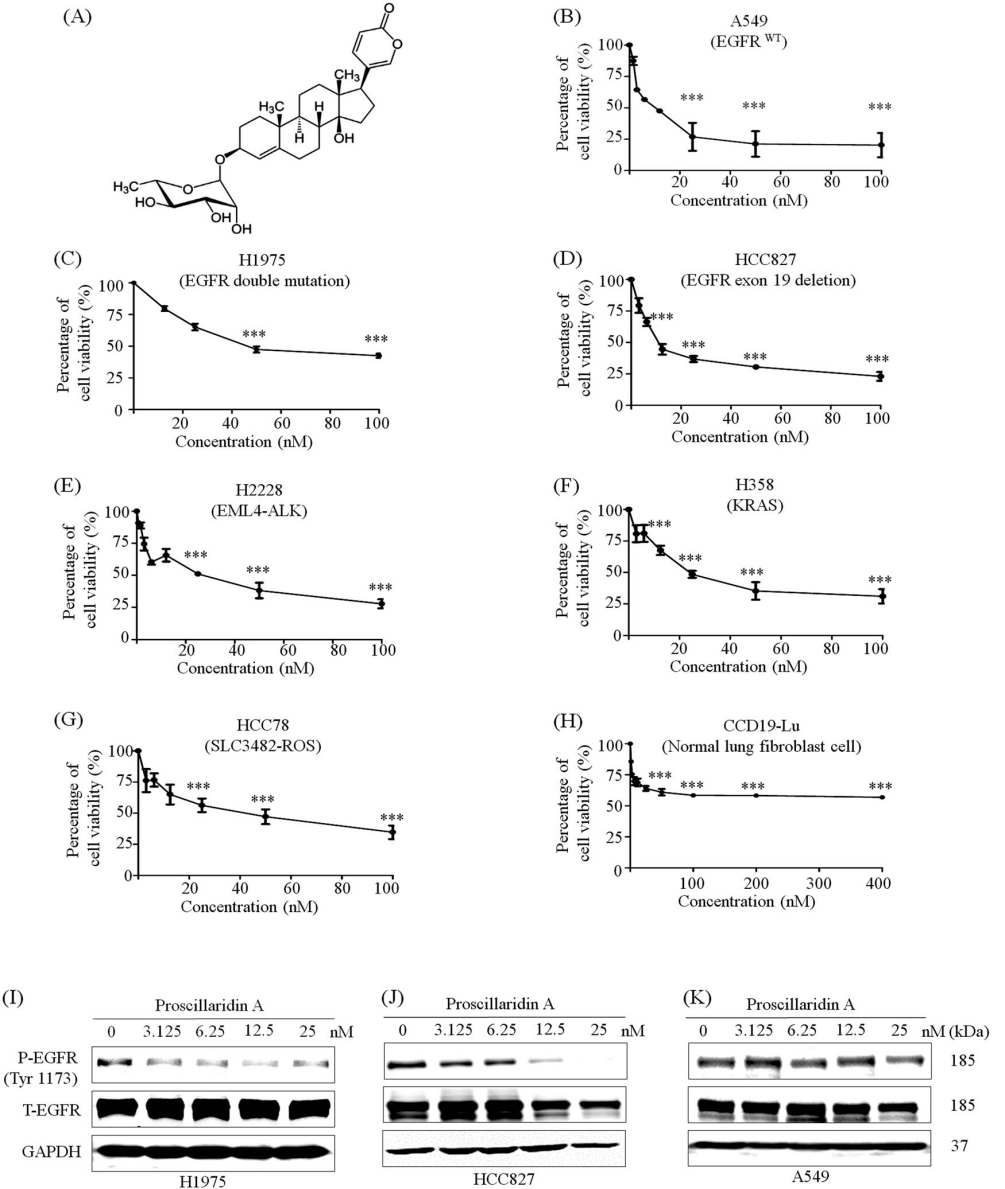
P.A shows significant cytotoxicity in NSCLC cells and inhibits EGFR phosphorylation in EGFR mutant cells. **a** The chemical structure of P.A; **b**–**h** P.A dose–response curves in NSCLC cell lines and a normal lung fibroblast cell line (CCD19-LU). The results are expressed as the mean ± S.E. (*p < 0.05, **p < 0.01, ***p < 0.001); **i**–**k** P.A specifically inhibited the phosphorylation of EGFR tyrosine residue 1173 in EGFR mutant NSCLC cells but had no effect on EGFR phosphorylation in EGFR wild-type NSCLC cells (A549)

To measure the cytotoxic effects of P.A, we examined cytotoxicity in a panel of NSCLC cells. Among these NSCLC cell lines, we included six different NSCLC cell lines with common NSCLC driver mutations, including EGFR, ALK, KRAS, and ROS (see Supplementary Table 1). The cytotoxic effect of P.A on normal cells was also determined using a normal lung fibroblast cell line (CCD19-LU). As shown in Fig. 1a–h and Supplementary Table 1, P.A showed cytotoxic effects in NSCLC cells at a range of 5–30 nM. Interestingly, the cell viability of normal lung fibroblast cells (CCD19-LU) was the highest after P.A treatment. Even using a concentration 30-fold higher than the CC_50_ value for the EGFR mutant NSCLC cells (H1975 and HCC827) to treat CCD19-LU, there was still no significant inhibitory effect, implying low toxicity of P.A in normal lung fibroblast cells. P.A showed significant cytotoxicity in NSCLC cells and low cytotoxicity in normal cells, which may be advantageous for future pharmaceutical applications.

### P.A induces apoptosis in NSCLC cells and suppresses EGFR activation in EGFR mutant cell lines

**Fig. 2:**
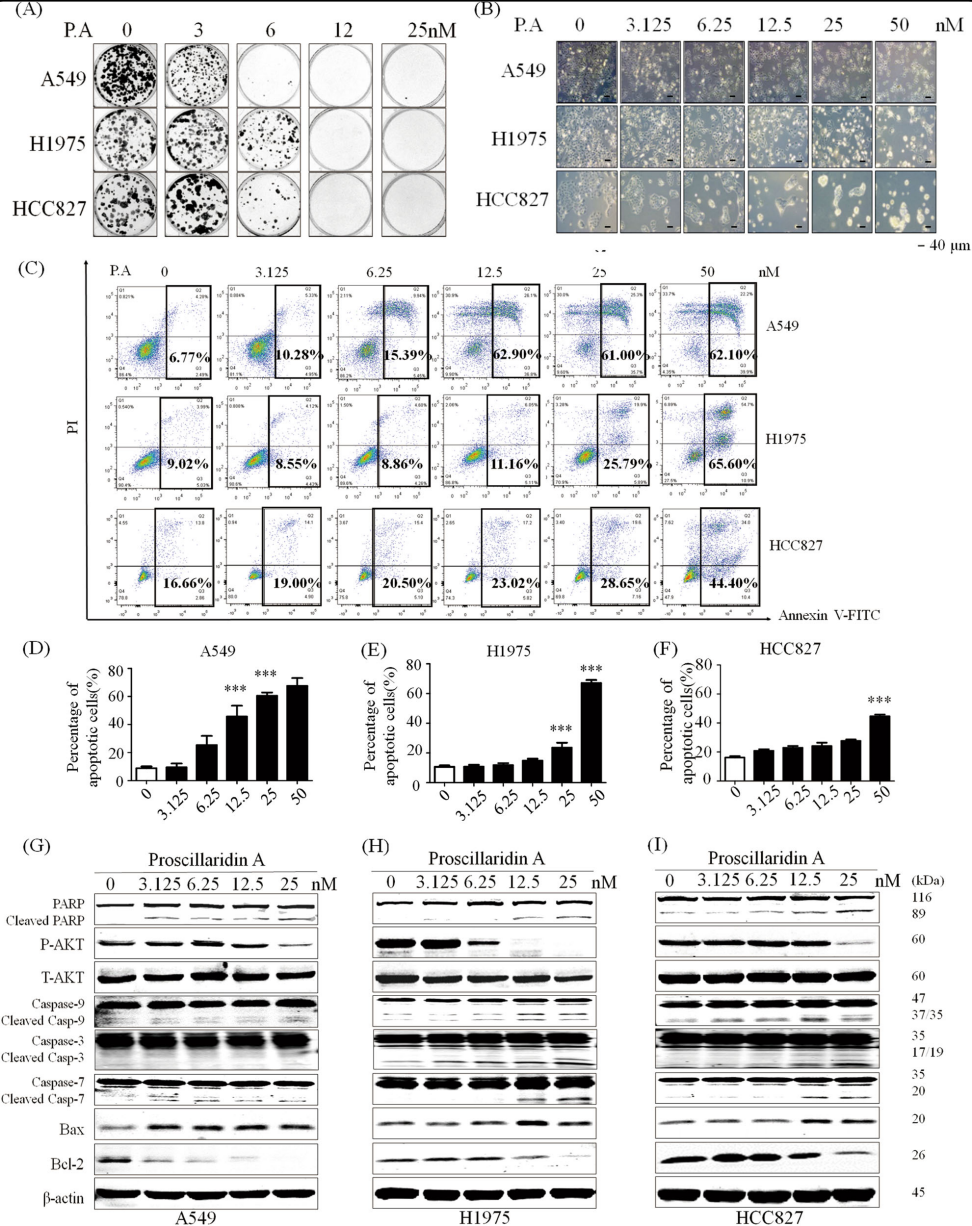
P.A inhibits colony formation and significantly induces apoptosis in NSCLC cells. **a** Data are shown as representative photomicrographs from colony formation assays after treatment of three NSCLC cell lines with different concentrations of P.A; **b** Morphological changes in three NSCLC cell lines after treatment with P.A; **c** Flow cytometric analysis of apoptosis levels after P.A treatment and statistical analysis results; **d**–**f** Statistical results of C (three independent experiments); **g**–**i** PARP, Caspase-3, Caspase-7, Caspase-9, and Bax were cleaved and activated by P.A, while Bcl-2 was downregulated and Akt activation was inhibited (*p < 0.05, **p < 0.01, ***p < 0.001)

Since P.A could inhibit cell proliferation in NSCLC cells harboring different common driver gene mutations, the most sensitive thrgee cell lines (A549, H1975, and HCC827) were chosen for treatment mechanism studies. As H1975 and HCC827 harbor an EGFR mutation and A549 is EGFR wild type, we compared the effects of P.A on EGFR activity among EGFR mutant and wild-type cells by measuring EGFR phosphorylation levels. The results showed that P.A inhibited the phosphorylation of EGFR at tyrosine residue 1173 in EGFR mutant cell lines (H1975 and HCC827) but not in the EGFR wild-type cell line (A549) (Fig. 1i–k). Next, we were interested in investigating whether the treatment of P.A affected the ability of cells to form colonies. The results demonstrated that, although EGFR was inhibited to different degrees in these cell lines, P.A could suppress clonogenic growth in all three NSCLC cell lines (Fig. 2a). Thus, the results demonstrated that P.A induced cell death through independent pathways.

To determine how P.A suppressed cell proliferation in NSCLC cells of different EGFR mutational statuses, we examined the apoptotic effect of P.A in A549, H1975 and HCC827 by microscopic and flow cytometric analyses. As shown in Fig. 2b, c, P.A significantly induced apoptosis in A549 and H1975 cells beginning at 12.5 and 25 nM, respectively, and induced apoptosis in approximately 70% of cells at 50 nM. In HCC827, P.A only induced apoptosis in ~30% of cells at 50 nM. Furthermore, as shown in Fig. 2g–i, PARP, Caspase-3, Caspase-7, and Caspase-9 were cleaved and activated by P.A treatment. The pro-survival regulator Akt was inhibited and the pro-apoptotic protein Bax was upregulated, while the anti-apoptotic protein Bcl-2 was downregulated, indicating that P.A-induced cell death was mediated through apoptosis. We further applied BAX immunofluorescence (IF) staining and mitochondrial extracted protein WB analysis to examine the localization of BAX after the treatment. From the results (supplementary Figure 5), we could observe the increased BAX fluorescence and upregulated BAX protein level in mitochondrial. To better understand the time-dependent cell death mechanisms, we tested P.A efficacy in different time points which exhibited the time-dependent manner of P.A (supplementary Figure 1-2). After comparing the P.A sensitivity results, the P.A-sensitive A549 and H1975 cells were chosen for subsequent functional assays.

### P.A significantly inhibits Na^+^/K^+^ ATPase activity and increases Ca^2+^ levels, contributing to its cytotoxicity in cancer cells

**Fig. 3:**
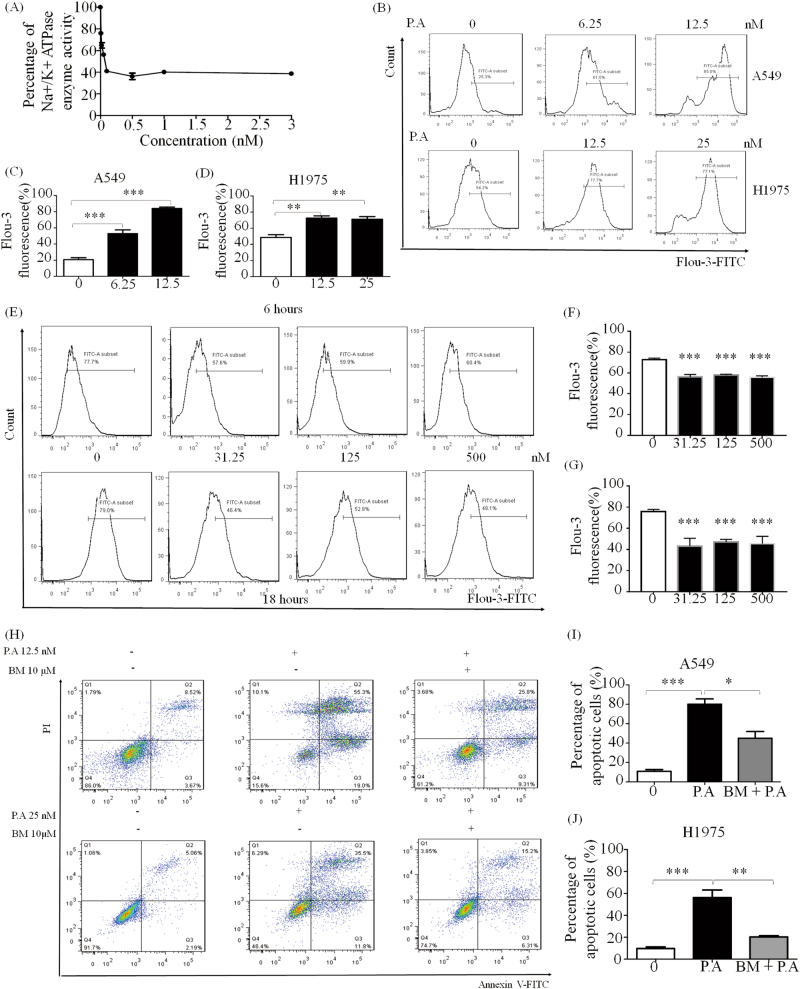
P.A inhibited Na^+^/K^+^ ATPase and regulated [Ca^2+^] levels in NSCLC. **a** P.A significantly inhibited Na^+^/K^+^ ATPase, and its IC_50_ was measured by in vitro Na^+^/K^+^ ATPase assay; **b** P.A elevated [Ca^2+^] levels after 6 h of treatment in A549 and H1975 cells; **c**, **d** Statistical results of **b** (three independent experiments); **e** P.A decreased [Ca^2+^] levels after 6 and 18 h treatment in CCD19-LU; **f**, **g** Statistical results of **e** (three independent experiments); **h** Increased intracellular [Ca^2+^] levels were required for P.A-induced apoptosis. A calcium chelator (BM) remarkably inhibited P.A-induced apoptosis; **i**, **j** Statistical results of **e** (three independent experiments) (*p < 0.05, **p < 0.01, ***p < 0.0001)

To investigate whether P.A could inhibit the Na^+^/K^+^ ATPase, enzyme activity was assessed by in vitro Na^+^/K^+^ ATPase assay. As shown in Fig. 3a, the EC_50_ was 0.83 ± 0.3 nM, which is very potent, implying that P.A could directly inhibit the activity of Na^+^/K^+^ ATPase. As P.A inhibits Na^+^/K^+^ ATPase, it follows that ion levels would change^23,24^. Thus, we investigated whether inhibition of Na^+^/K^+^ ATPase would stimulate the Na^+^/Ca^2+^ exchanger, which would induce an increase in Ca^2+^ levels. As shown in Fig. 3b, P.A significantly increased intracellular Ca^2+^ levels at 12.5 nM in A549 and H1975 cells. We also tested the unfolded protein response after the treatment of P.A. In the results, the expression of BIP (GRP78) showed dosage-dependent decrease (supplementary Figure 6). However, in the normal lung fibroblast cell line, CCD19-LU, P.A treatment decreased Ca^2+^ levels, which may explain why CCD19-LU had the highest P.A CC_50_ among all the cell lines. The results also demonstrated that P.A treatment remarkably increased in vitro Ca^2+^ levels.

To ascertain whether Ca^2+^ elevation was important to P.A-induced apoptosis, the Ca^2+^ chelator BAPTA/AM (BM), which can decrease in vitro Ca^2+^ levels, was applied in conjunction with P.A. As shown by the flow cytometric results, the number of apoptotic cells significantly decreased following co-treatment with BM and P.A (Fig. 3h). The single treatment cytotoxicity of BM was shown in supplementary Figure 3. These results suggested that elevation of Ca^2+^ levels is a key mediator of P.A-induced apoptosis.

### P.A activates AMPK and JNK phosphorylation and downregulates ACC phosphorylation

**Fig. 4:**
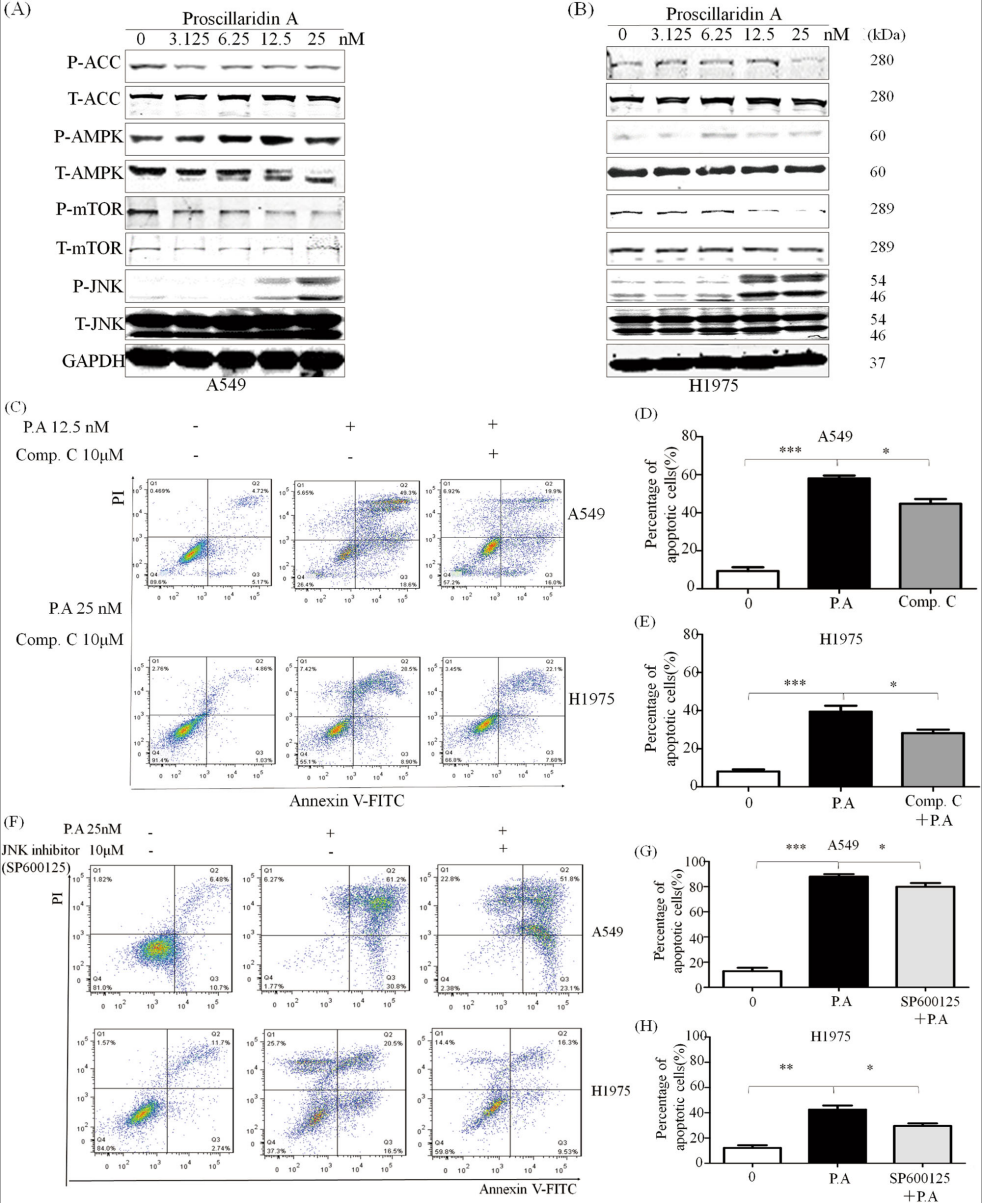
P.A activated the AMPK pathway. **a**, **b** P.A increased the phosphorylation of AMPK and its downstream target ACC; **c** Inhibition of AMPK by compound C partially rescued the cells from apoptosis; **d**, **e** Statistical results of C (three independent experiments); **f** The JNK inhibitor decreased apoptosis in both cell lines; **g**, **h** Statistical results of **f** (three independent experiments)

It is well known that the induction of calcium influx is one of the most important AMPK activation mechanisms^25,26^. Since P.A could elevate Ca^2+^ levels in vitro, we investigated whether P.A could induce activation of AMPK. As shown in Fig. 4a, b, P.A induced the phosphorylation of AMPK and down-regulated its direct downstream target acetyl-CoA carboxylase (ACC). To investigate whether the activation of AMPK was important for P.A-induced apoptosis, we applied compound C, a specific inhibitor of AMPK, to block the activation of AMPK. Our results demonstrated that compound C partially reduced the number of apoptotic cells in both the A549 and H1975 cell lines (Fig. 4c). Thus, AMPK is also an important modulator of P.A-induced apoptosis.

AMPK is a major upstream regulator of the mTOR pathway and AMPK activation can suppress the activity of the mTOR pathway^27^. Thus, we further investigated whether P.A could inhibit mTOR phosphorylation. As shown in Fig. 4a, P.A indeed inhibited the phosphorylation of mTOR. Moreover, c-Jun N-terminal kinase (JNK), which is closely associated with the mTOR pathway, was also shown to be involved in P.A-induced apoptosis. As shown in Fig. 4a, P.A significantly activated the phosphorylation of JNK. To ascertain whether the activation of JNK was required for P.A-induced apoptosis, we utilized the JNK specific inhibitor SP600125. As shown in Fig. 4f, JNK inhibition reduced the number of apoptotic cells when used in conjunction with P.A, indicating that the JNK pathway also contributes to P.A-induced apoptosis. The single treatment cytotoxicity of compound C and SP600125 was shown in supplementary Figure 3.

### P.A upregulates DR4 expression and inhibits the DR4-suppressive NF-κB pathway

**Fig. 5:**
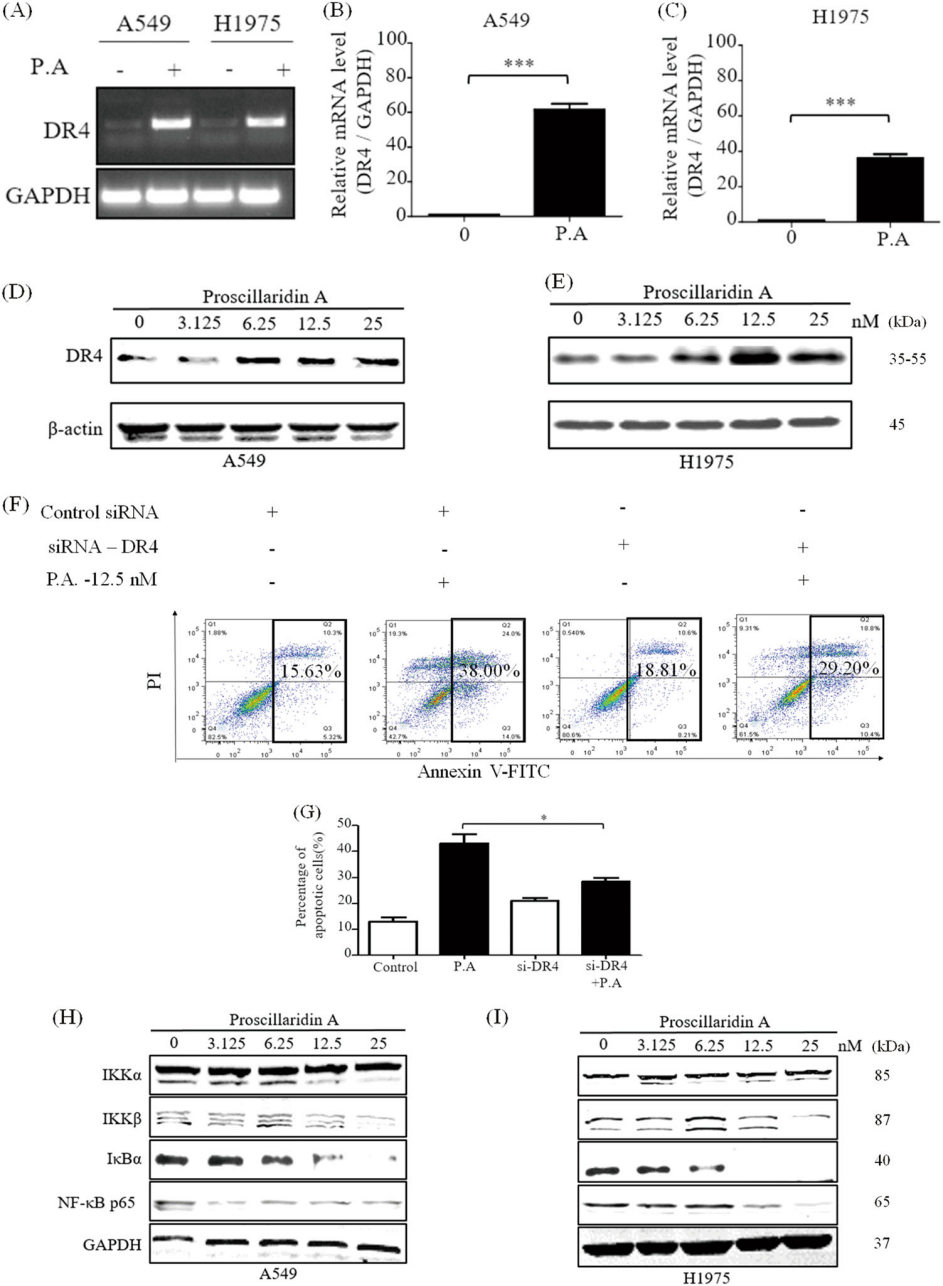
P.A increased death receptor 4 (DR4) expression. **a** Standard and **b**, **c** quantitative RT-PCR were performed to determine the expression of DR4 after a 12-h P.A treatment; **d**, **e** DR4 and its downstream effector, Caspase-8, were upregulated by P.A; **f** A549 cells were transfected with si-DR4 and treated with or without P.A for the indicated amount of time. Cells were collected and analyzed by flow cytometry with PI and ANNEXIN V; **g** Statistical results of **f** (three independent experiments); **h**, **i** The activation of the NF-κB pathway was inhibited by P.A treatment

The Ca^2+^-regulated tumor necrosis factor (TNF) receptor is closely related to the apoptotic mechanism^28,29^. The TNF-related apoptosis-inducing ligand (TRAIL) can induce tumor cell death by interacting with TRAIL-receptor 1, also known as death receptor 4 (DR4)^30,31^. We investigated DR4 gene expression following treatment with P.A. As shown in Fig. 5a–c, both standard and quantitative PCR results demonstrated that the expression of DR4 was significantly elevated following P.A treatment. The protein level results were consistent with the gene expression results (Fig. 5d, e). In addition, when DR4 was knocked down, the percentage of apoptotic cells decreased (Fig. 5f). This may indicate that DR4 is an important mediator of P.A-induced apoptosis. WB was applied to confirm the efficacy of siRNA used to knockdown DR4 and the results supported it (supplementary Figure 4). Subsequently, NF-κB, a suppressor of DR4^32^, has been shown to bind to the DR4 promoter at an NF-κB binding site (−1366/1356)^33^. NF-κB has also been shown to be downstream of mTOR pathway^34^. The results showed that the NF-κB pathway was significantly inhibited by P.A in a dose-dependent manner (Fig. 5h, i). This suggests that NF-κB signaling pathways could also be affected by P.A treatment.

### P.A suppresses tumor growth in H1975 xenografts

**Fig. 6:**
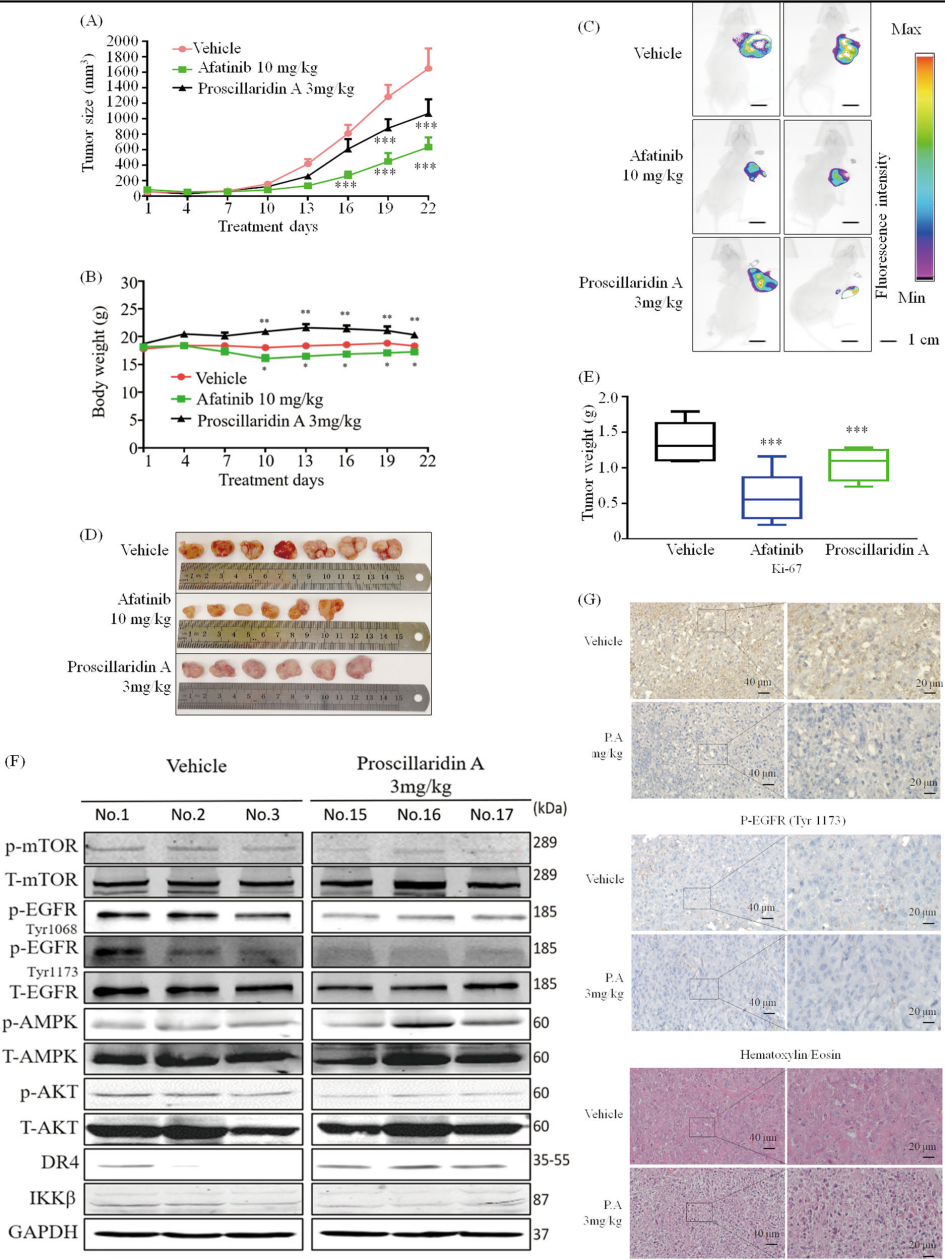
P.A suppresses tumor growth in H1975 xenografts. **a** Tumor volume was examined by caliper measurements from the beginning of the treatment period (when the tumor reached approximately 100 mm^3^ in size), *n* = 7 per group; **b** Quantification of body weight for each group; **c** Fluorescence intensity indicating the size of the tumor in vivo; **d**, **e** The weight of each tumor; **f** P.A decreased the phosphorylation of mTOR, EGFR (Tyr 1173 and Tyr 1068) and Akt activated AMPK and increased DR4 expression; **g** Immunohistochemistry analysis comparing P.A-treated tumor tissue to vehicle-treated tissue (*p < 0.05, **p < 0.01, ***p < 0.001)

To further understand the pharmaceutical activity of P.A, we next investigated its in vivo efficacy. GFP-transformed H1975 stable cells were injected into nude mice, and the tumors were allowed to grow to ~100 mm^3^ in size before beginning 21 days of P.A treatment (7 mice in each group). As shown in Fig. 6a, c, tumor growth was significantly suppressed after 16 days in the P.A group compared to the vehicle group. On the last day of treatment, the net tumor mass was measured and the average tumor weight in the P.A treatment group was 35% less than the average tumor weight in the vehicle group (Fig. 6d, e). Mice in the P.A group displayed greater body weight compared to the vehicle group (the statistical analysis of body weight on their sacrificed day was shown in supplementary Figure 9). Compared to the Afatinib group, mice in the P.A group showed no apparent toxicity after treatment (Fig. 6b). We further examined the effect of P.A treatment on EGFR, AMPK, mTOR, Akt, and death receptor cascades in protein extracts derived from tumor tissues. Consistent with the in vitro data, we observed suppression of EGFR, Akt, and ACC phosphorylation and an increase of AMPK and DR4 (Fig. 6f). Moreover, as shown in Fig. 6g, immunohistochemistry analysis demonstrated that P.A treatment decreased EGFR phosphorylation (Tyr 1173) in tumor cells and notably reduced cell proliferation, as indicated by Ki-67 immunostaining. P.A also decreased cellularity, as measured by hematoxylin and eosin staining. Thus, these data demonstrate that P.A is detrimental to NSCLC tumor growth in preclinical lung xenograft models.

## Discussion

Recent reports have indicated that steroidal cardiac inhibitors could inhibit the in vivo growth of prostate and lung xenografts through significant up-regulation of caspase-3 activity^35^ and could induce apoptosis in glioblastoma cells through activation of the p38 and JNK pathways^36^. Therefore, Na^+^/K^+^ ATPase is an emerging target in anti-cancer therapy^37^ and is currently being investigated for the development of novel therapeutics^38^. The balance of cellular membrane potential is regulated by P-type ATPase family pumps, including Na^+^/K^+^ ATPase and Na^+^/Ca^2+^ exchanger^39^. These ion pumps are closely linked. For example, if Na^+^/K^+^ ATPase activity is interrupted, cellular Na^+^/K^+^ homeostasis and, subsequently, other ionic gradients such as Ca^2+^ will also be disrupted^11^. As a second messenger, Ca^2+^ is involved in various fundamental functions, including suppressing Bcl-2 expression and activating the caspase pathway^24^ to induce apoptosis^40^ and participating in Ca^2+^/calmodulin-dependent kinase kinase β (CaMKKβ)-mediated AMPK activation and other various metabolic pathways^41–44^.

**Fig. 7:**
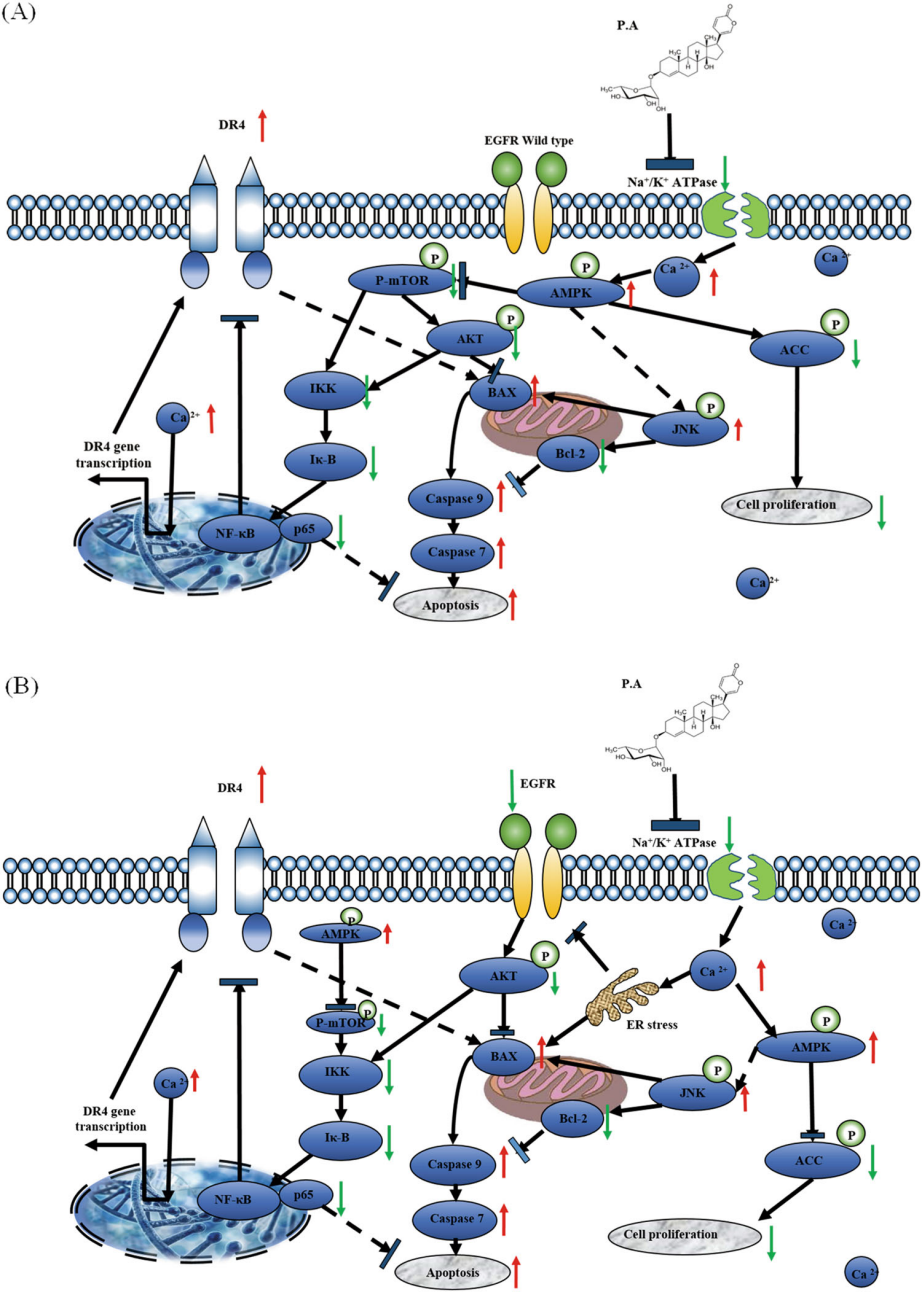
The proposed mechanism of action of P.A in NSCLC cells

In this study, we have found that P.A has anti-cancer effects through its ability to inhibit Na^+^/K^+^ ATPase. As shown in Fig. 7a, P.A inhibits Na^+^/K^+^ ATPase and elevates Ca^2+^ levels, resulting in the activation of the AMPK pathway. More interestingly, after an 18-hour P.A treatment in the normal lung fibroblast cell line CCD19-LU, Ca^2+^ levels decreased by almost 30%. Other investigators have demonstrated that cardiac glycosides have distinct effects on the regulation of Ca^2+^ levels and apoptosis in human fibroblasts and cancer cells, which is in line with the results of our study^45^. Compared with other cardiac glycosides, the CC_50_ of P.A is 50% of that of Digoxin in A549s, almost 10% of that of Digoxin in H1975s^46^, and less than the CC_50_ of Digitoxin in both EGFR-related cell lines^18^. However, P.A. demonstrated less toxicity in normal cells than both Digoxin and Digitoxin. Activation of AMPK exerts apoptotic effects through two main pathways: suppression of ACC phosphorylation and hence mTOR signaling or stimulation of the JNK pathway. Moreover, elevations in Ca^2+^ levels can also induce ER stress^47^, which will inhibit EGFR phosphorylation^48,49^, thus elucidating the connection between Ca^2+^ and EGFR. As shown in EGFR mutant cells (Fig. 7b), P.A induces the elevation of Ca^2+^ levels, which further inhibits EGFR phosphorylation. Inhibition of EGFR also affects other apoptotic signaling pathways, for example by inactivating phosphor-Akt and activating the JNK pathway, both of which will further induce apoptosis.

High intracellular Ca^2+^ is not only related to inhibition of AMPK and EGFR but also inhibition of another important apoptotic cytokine, tumor necrosis factor (TNF). The TNF family is known to transduce death signals via cell membrane receptors. DR4, after binding to the apoptotic cytokine TRAIL, can recruit and activate the apoptosis-initiating protease caspase-8, which in turn activates ‘effector’ caspases, including caspase-3, -6, and -7, which execute the cell’s apoptotic demise^50,51^. Recent research shows that a combination of TRAIL and the herbal extraction apigenin (APG) or the chemotherapeutic drug *cis*-diaminedichloro- platinum (II) (CDDP) can induce apoptosis by upregulating the expression of DR4^52^ and DR5^53^ in different cancer cells. APG alone has low cytotoxicity in cancer cells. Compared to the reports above, P.A shows better treatment efficacy and enhances DR4 expression at a nanomolar level and the combination treatment with TRAIL showed significantly additive effects, increased nearly 20% apoptosis at 12.5 nM with 25 ng/ml TRAIL subtract both single treatment apoptotic percentage (supplementary Figure 7-8).

NF-κB, a suppressor of the DR4 pathway, is one of the most important signal transduction pathways and exhibits the ability to promote cell proliferation, suppress apoptosis, promote cell migration, and suppress differentiation^54^. It has been reported that a hemi-synthetic cardiac inhibitor, UNBS1450, can induce apoptotic cell death by inhibiting NF-κB transactivation. Recent studies have also demonstrated that TRAIL-mediated cell death is enhanced upon infection of neuroblastoma cells with a dominant-negative mutant of IκB kinase, a kinase essential for NF-κB activation^32^. P.A inhibits the activation of IKKβ, IκBα, and NF-κB through regulation of AMPK, the mTOR cascade and the EGFR and Akt pathways.

The significance and novelty of this study lie in the identification of an effective Na^+^/K^+^ ATPase inhibitor, P.A, which can significantly inhibit the growth of NSCLC cells via Ca^2+^-induced DR4 up-regulation at the nanomolar level. This is the first demonstration of the connection between Ca^2+^ gradients, DR4 and apoptotic signaling pathways in NSCLC cells following P.A treatment. This compound also shows low toxicity in normal lung fibroblast cells, demonstrating its promise for further development as a new anti-cancer drug. Though there have been studies using P.A in glioblastoma^22^, breast cancer^20,21^, and colon cancer^55^, the compound was less effective in these tumor types than in NSCLC cells. Further, the anti-cancer mechanisms demonstrated by other studies were shown through only one angle, such as the inhibition of HIF-1α^19^ or the inhibition of topoisomerase I and II^20,21^. We, for the first time, demonstrate how P.A can function through a combination of AMPK activation and EGFR inhibition, resulting in a potent anti-cancer effect.

In summary, we provide new evidence that P.A is useful in the treatment of EGFR-mutant NSCLC cell lines and displays a different treatment mechanism in these cell lines compared with non-EGFR-mutant NSCLC cell lines. P.A can elevate the expression of DR4 and inhibit its suppressor, NF-κB, which is a key mediator of tumor cell survival. P.A also has the ability to suppress tumor growth in vivo. We believe that P.A is a promising candidate for treating NSCLC in the future.

## Materials and methods

### Cell culture and reagents

All cell lines used in this study were purchased from ATCC (American Type Culture Collection, Manassas, VA, USA). Proscillaridin A was purchased from Sigma (St Louis, MO, USA). Primary antibodies were purchased from Cell Signaling Technology (Danvers, MA, USA). The fluorescein-conjugated anti-rabbit secondary antibody was purchased from Odyssey (Belfast, ME, USA).

### MTT assay

Cells were seeded in a 96-well microplate with 3000–5000 cells/well and incubated overnight to allow cells to adhere. Cells were treated with different concentrations of P.A or the vehicle control dimethyl sulfoxide (DMSO). Ten microliters of MTT (5 mg/ml) solution was added to each well for 4 h. After that, 100 μL of resolved solution (10% SDS and 0.1 mM HCL) was added to each well for another 4 h. Absorbance was measured at 570 nm (with a reference reading at 650 nm) on a microplate reader (Tecan, Morrisville, NC, USA).

### Colony-formation assay

Colony-forming assays were performed as previously described^18^. Briefly, approximately 300 cells were plated into each well of a 6-well plate. After 48 h, cultures were rinsed with fresh medium and P.A (with different indicated dosages) was added to the medium. Forty-eight hours later, the cells were washed twice with PBS and then incubated in drug-free medium. The medium was changed every 5 days. After culturing for an additional 10–14 days, the cells were fixed with 3.7% paraformaldehyde for 20 min and incubated with 0.2% crystal violet solution in 10% ethanol for 10 min.

### Apoptosis assay

A549, H1975, and HCC827 cells (1 × 10^5^ cells/well) were seeded in a six-well plate for 24 h and then treated with the indicated concentrations of P.A for an additional 24 h at 37 °C. The cells were washed once with ice-cold 1× PBS and then harvested. Then, the cell pellets were re-suspended in 100 μl of 1× binding buffer. The cells were double-stained with 2 μl each of Annexin-V FITC and PI (100 μg/ml) for 15 min at room temperature (RT) in the dark. Apoptotic cells were quantified on a BD Aria III Flow Cytometer (BD Biosciences, San Jose, California, USA)

### Western blot analysis

After incubating A549, H1975, and HCC827 cells with P.A for the indicated amount of time, cells were harvested and washed with cold 1× PBS. Then, cells were lysed with ice-cold RIPA lysis buffer containing protease and phosphatase inhibitors to extract the cellular proteins. Total extracted protein was quantified using the Bio-Rad DCTM protein assay kit (Bio-Rad, Philadelphia, PA, USA). Then, 35 μg of each protein sample was loaded and electrophoretically separated on an 8% SDS-PAGE gel and then transferred to a Nitrocellulose (NC) membrane. Membranes were blocked with 5% non-fat milk and PBS containing 0.1% Tween-20 (TBST) for 1 h at RT. After 1 h, membranes were incubated with primary antibodies (1:1000 dilution) at 4 °C overnight with gentle shaking. Membranes were washed three times with TBST (5 min/time) and incubated with a secondary fluorescent antibody (1:10,000 dilution) for 1 h at RT. After another three washes with TBST (15 min/time), bands were visualized on a LI-COR Odessy scanner (Belfast, ME, USA).

### Na^+^/K^+^ ATPase enzyme activity assay

The enzymatic activity of Na^+^/K^+^ ATPase (purchased from Sigma as a lyophilized powder from porcine cerebral cortex) was measured by colorimetric quantification of _Pi_ released during ATP hydrolysis. First, 10 μl of Na^+^/K^+^ ATPase (600 units/ml) was incubated with 2.5 μl of KCl/NaCl solution (45 mM KCl and 2 M NaCl) and either 5 μl of DMSO (control) or 5 μl of P.A in 67.5 μl of buffer (24 mM Tris HCl buffer with 0.68 mM ethylenediaminetetraacetic acid and 6.0 mM magnesium chloride, pH 7.8). ATP (5 μl of 80 mM solution) was then added. Trichloroacetic acid (30 μl of 100% w/v) was added to the reaction, then the mixture was centrifuged for 5 min. The supernatant (50 μl aliquot) was transferred to a 96-well plate containing 100 μl of Taussky–Shorr reagent. Following a 5 min incubation at RT, absorbance was read at 660 nm.

### Measurement of intracellular calcium

Changes in intracellular free calcium were measured using a fluorescent dye, Fluo-3, as previously described^56^. Briefly, A549 and H1975 cells were washed twice with culture media after a 6-h P.A treatment (6.25–25 nM). Then, cell suspensions were incubated with 5 μM Fluo-3 at 37 °C for 30 min. The cells were then washed twice with HBSS and the re-suspended cell samples were subjected to FACS analysis. At least 10,000 events were analyzed

### RNA extraction and quantitative real-time PCR

Cells were incubated with P.A for 12 h. Total RNA was then extracted from treated cells using a TRIzol reagent (Invitrogen, Carlsbad, CA, USA). Quantitative real-time PCR was performed using High-productivity Real-Time quantitative PCR ViiA^TM^7 (Life Technologies, Gaithersburg, MD, USA). The PCR primers used in our study were synthesized commercially, and the sequences are as follows: DR4: 5′- TTGTGTCCACCAGGATCTCA-3′ and 5′-GTCACTCCAGGGCGTACAAT-3′. The glyceraldehyde 3-phosphatase dehydrogenase (GAPDH) gene was used as the reference gene. GAPDH: 5′-AACGACCCCTTCATTGAC-3′; and 5′-TCCACGAC ATACTCAGCAC-3′. All data are represented as the mean fold change values normalized to GAPDH and obtained from triplicate analyses.

### Transfection with small interfering RNA

A549 cells were seeded into six-well plates. After 24 h, cells were transfected with DR4 or control small interfering RNAs (siRNAs) using 2 μl of X-tremeGENE siRNA transfection reagent (Roche, Basal, Switzerland). All siRNAs were synthesized by GenePharma (Pudong New Area, Shanghai, China). The sequences of the siRNAs used are as follows (sense and antisense, respectively): siDR4 5′-r(CAAACUUCAUGAUCAAUCA)dTdT-3′ and 5′-r(UGAUUGAUCAUGAAGUUUG)dAdT-3′, and control siRNA 5′-r(UUCUCCGAACGUGUCACGU)dTdT-3′ and 5′-r(ACGUGACA CGUUCGGAGAA)dTdT-3′. Eight hours after transfection, cells were stimulated with 12.5 nM P.A. Cells were harvested 24 h after stimulation for determination of apoptosis by flow cytometry.

### Xenograft study

All animals were acclimatized for 1 week. Six-week-old nude mice were injected with 5 × 10^6^ GFP-transformed H1975 stable cells mixed with growth factor reduced Matrigel (Becton Dickinson, Franklin Lakes). Xenografts were allowed to grow to a size of ~100 mm^3,^ then mice were randomized into the following three treatment groups: vehicle, Afatinib (10 mg/kg i.p.) and P.A (3 mg/kg i.p.). Mice were treated daily for 21 days and tumor volumes were measured every third day. In vivo tumor fluorescence intensity was measured using the In-Vivo XTREME Imager (Bruker BioSpin, Billerica, MA, USA). Histology and immunohistochemistry analyses were performed using the DAKO EnVision^TM^ + system (Dako, Glostrup, Denmark).

### Statistical analysis

All the data are presented as the mean ± SD of three individual experiments. Differences were analyzed by one-way ANOVA using Graph Pad Prism 5.

## Electronic supplementary material

Supplementary material

Supplemental figure legends
