# Supplementary material for: Proscillaridin A induces apoptosis and suppresses non-small-cell lung cancer tumor growth via calcium-induced DR4 upregulation
Source: Cell Death Dis. 2018 Jun 13;9(6):696. doi: 10.1038/s41419-018-0733-4 (PMC5999972; doi:10.1038/s41419-018-0733-4)

NSCLC Cell lines	HCC827	A549	H1975
Mutation	EGFR exon 19 deletion	EGFR wild type	EGFR double mutant
Gefitinib sensitive	Sensitive	Sensitive	Resistant
CC_{50} (nM)	13.9 ± 1.8	14.0 ± 2.8	15.0 ± 3.6
NSCLC Cell lines	H2228	H358	HCC78
Mutation	EML4 - ALK	KRAS	SLC3482 – ROS
CC_{50} (nM)	23.6 ± 5.4	25.8 ± 4.9	37.6 ± 9.9
Normal Cell lines	CCD19-LU		
Derivation	Fibroblast cell		
CC_{50} (nM)	> 400		

Supplementary Table 1. The CC_{50} value of P.A on NSCLC cell lines and CCD19-LU normal lung fibroblast cell line

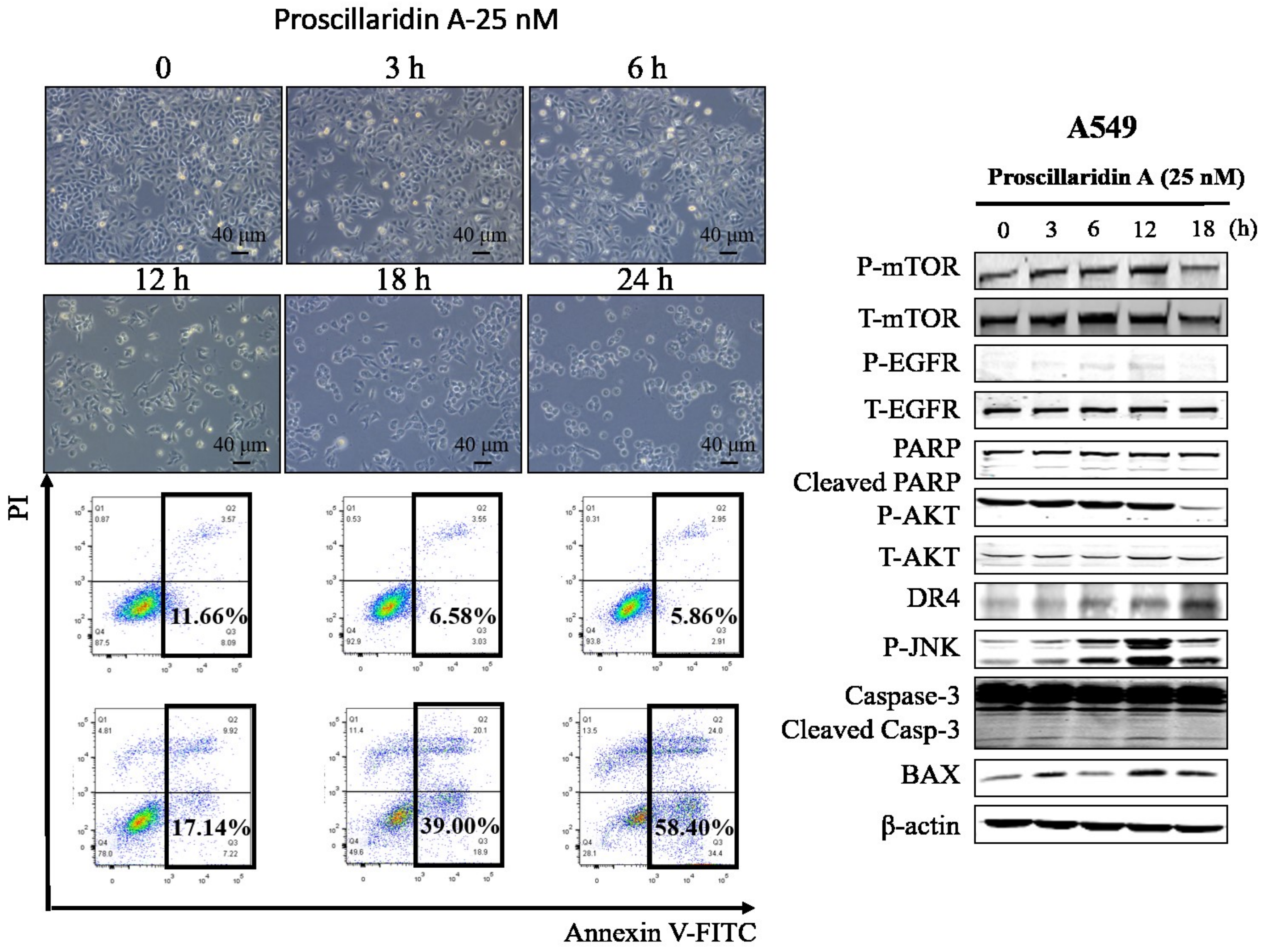

Supplementary Figure 1. The treatment effect of P.A at different time points in A549 cell line.

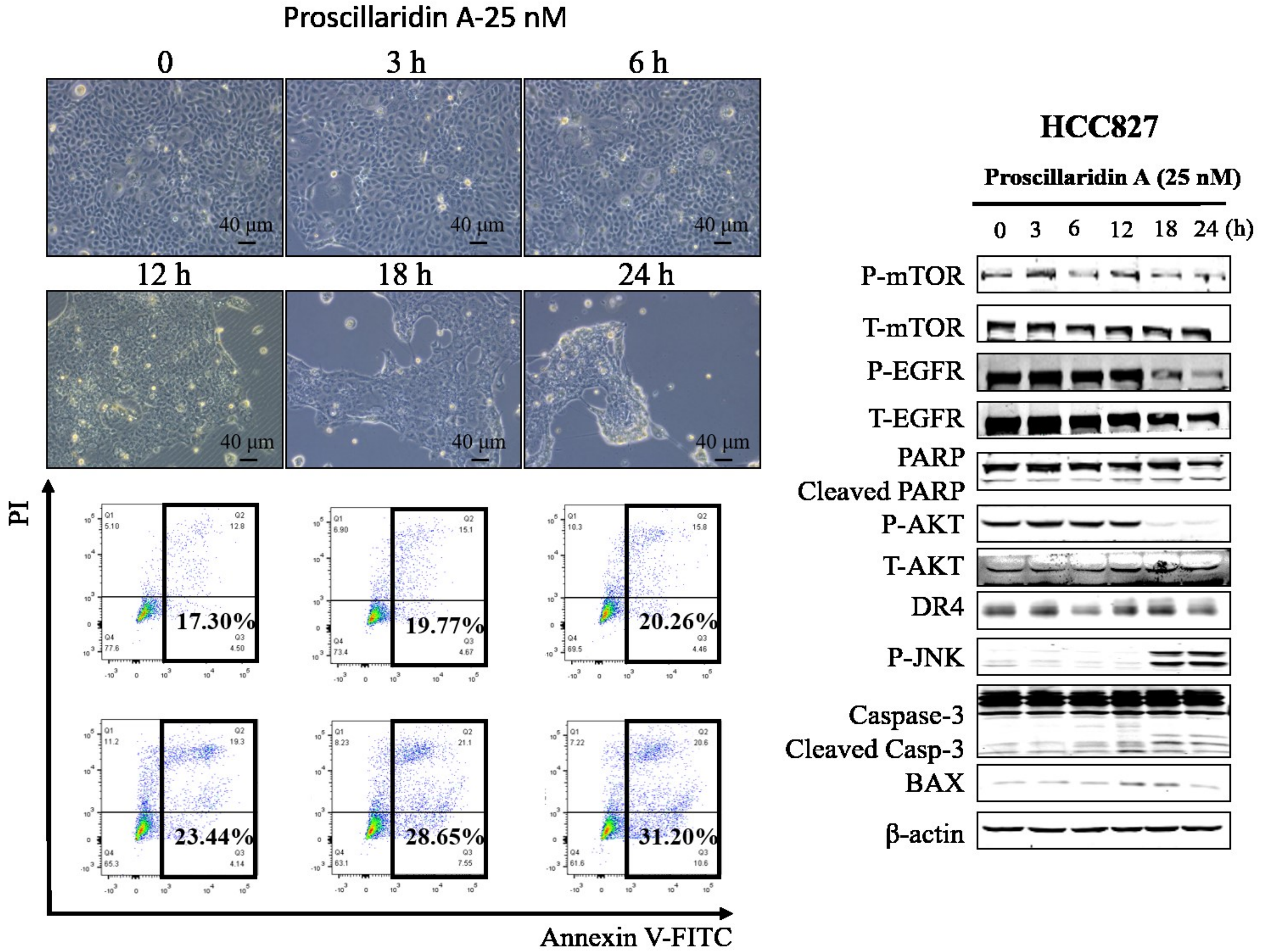

Supplementary Figure 2. The treatment effect of P.A at different time points in H1975 cell line.

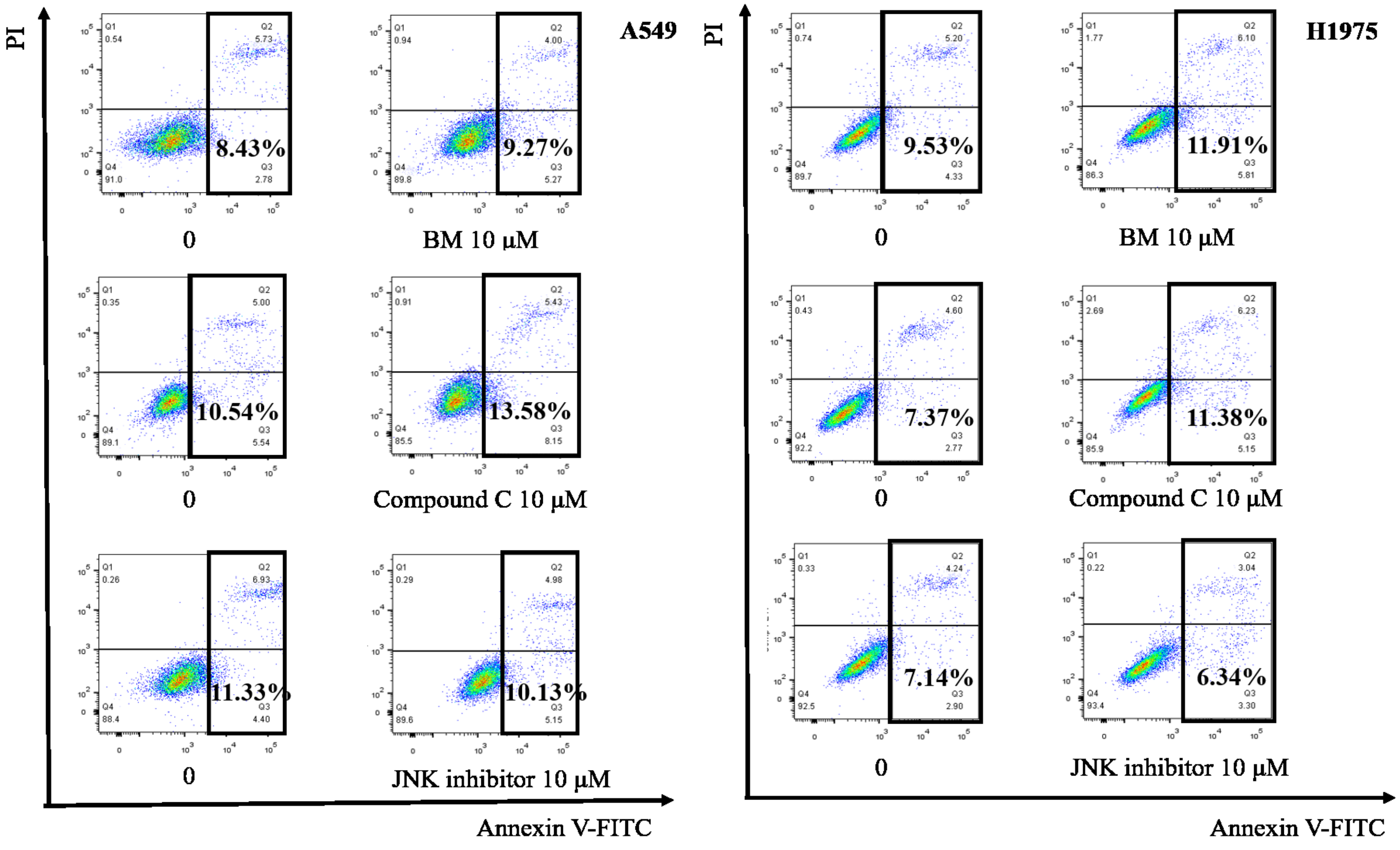

Supplementary Figure 3. The flow cytometry analysis of the cytotoxicity effect of BM, Compound C and JNK inhibitor.

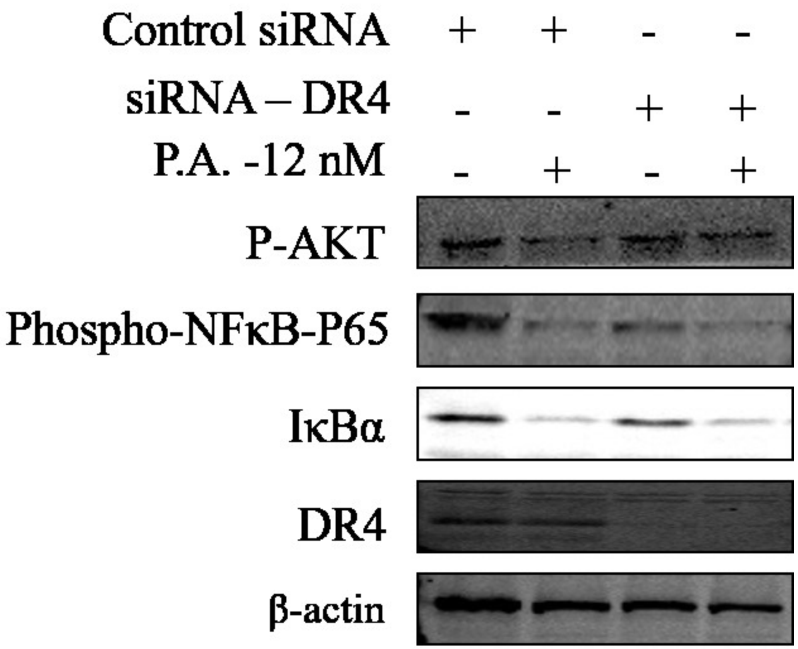

Supplementary Figure 4. The effect of DR4 siRNA knockdown.

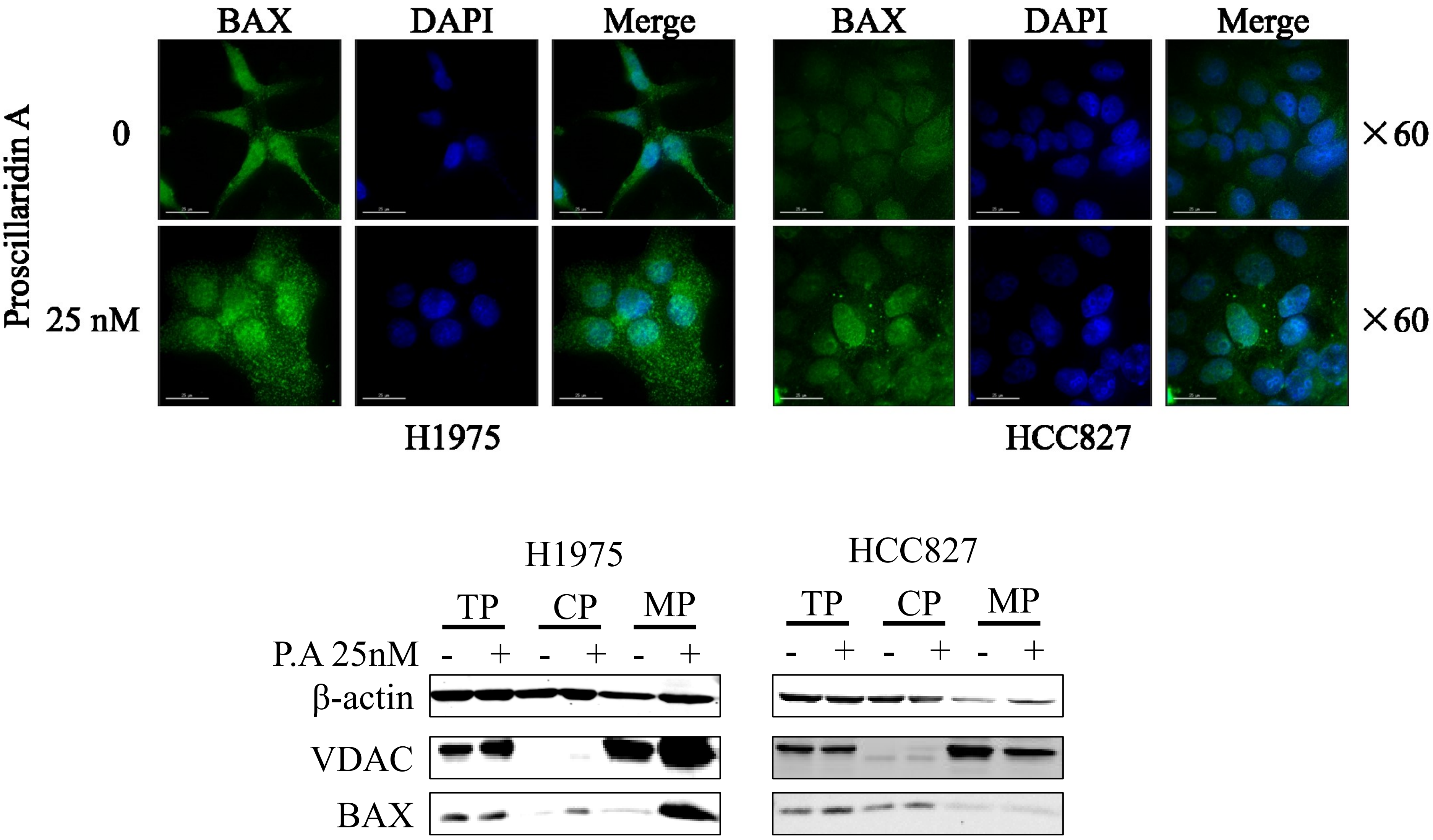

TP: Total protein; CP: Cytosol protein; MP: mitochondrial protein.

Supplementary Figure 5. The localization of BAX after the treatment of P.A

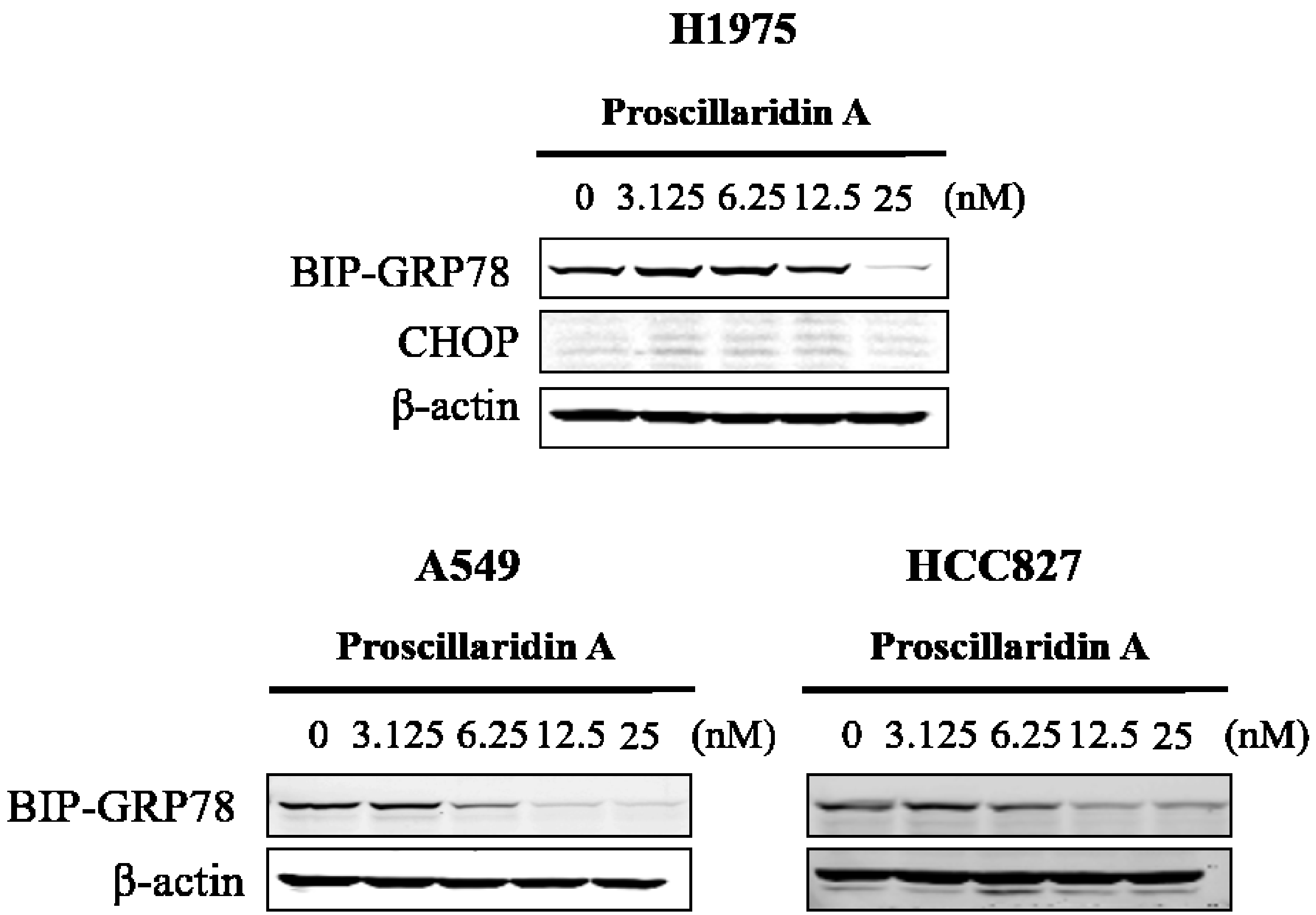

Supplementary Figure 6. The effect of P.A on the unfolded protein response.

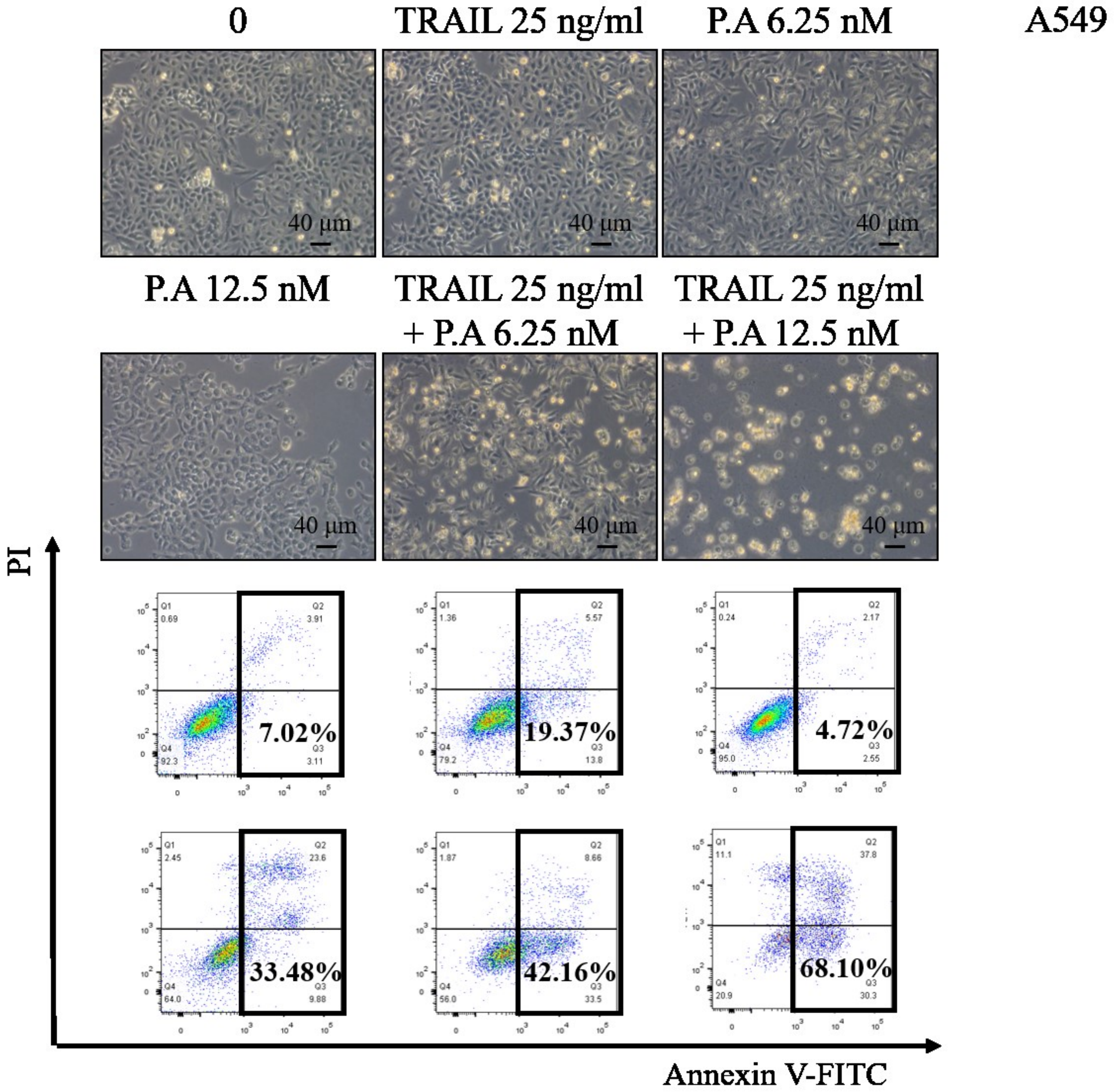

Supplementary Figure 7. The combination treatment effect of P.A and DR4 ligands in A549 cell line.

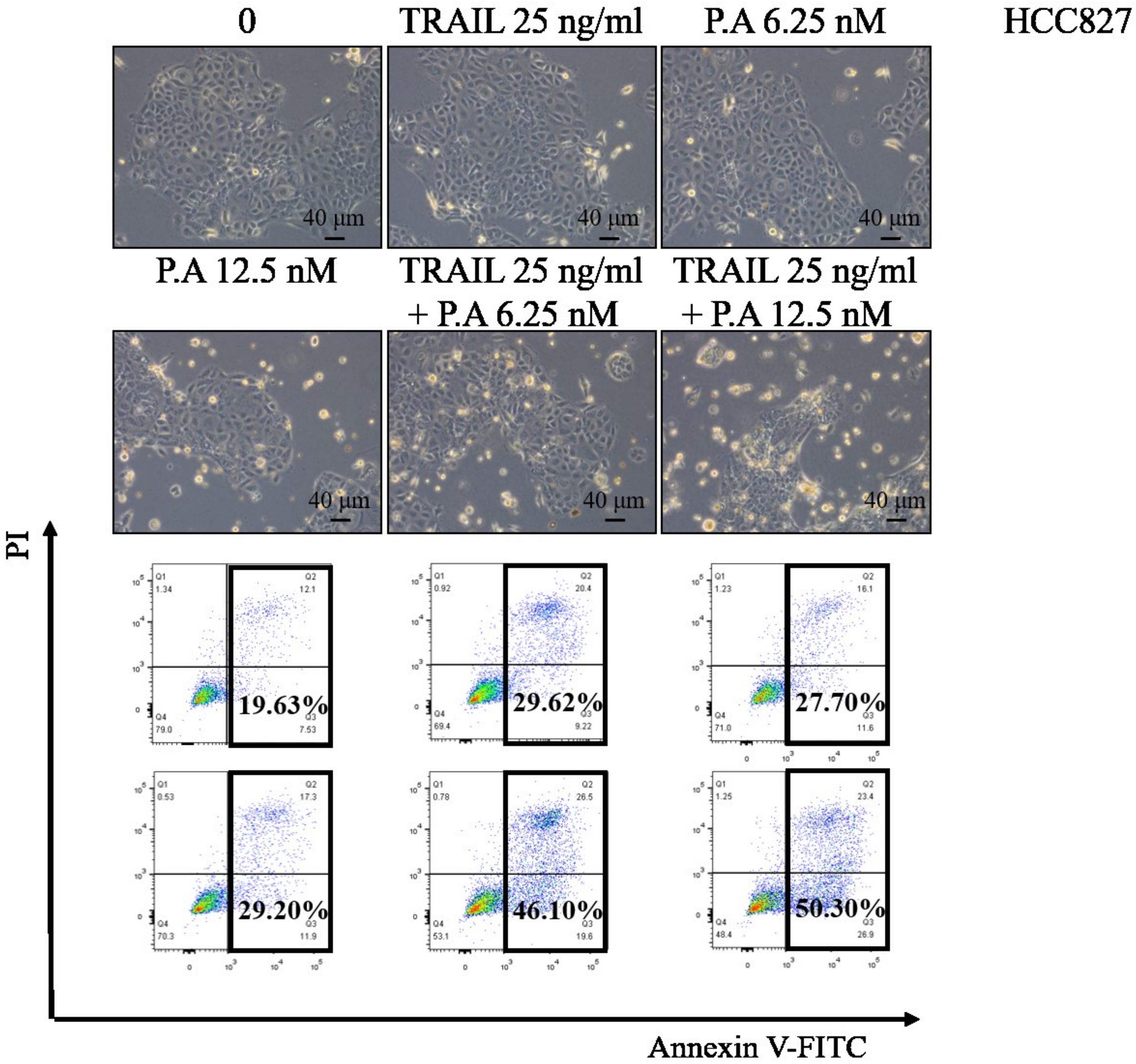

Supplementary Figure 8. The combination treatment effect of P.A and DR4 ligands in HCC827 cell line.

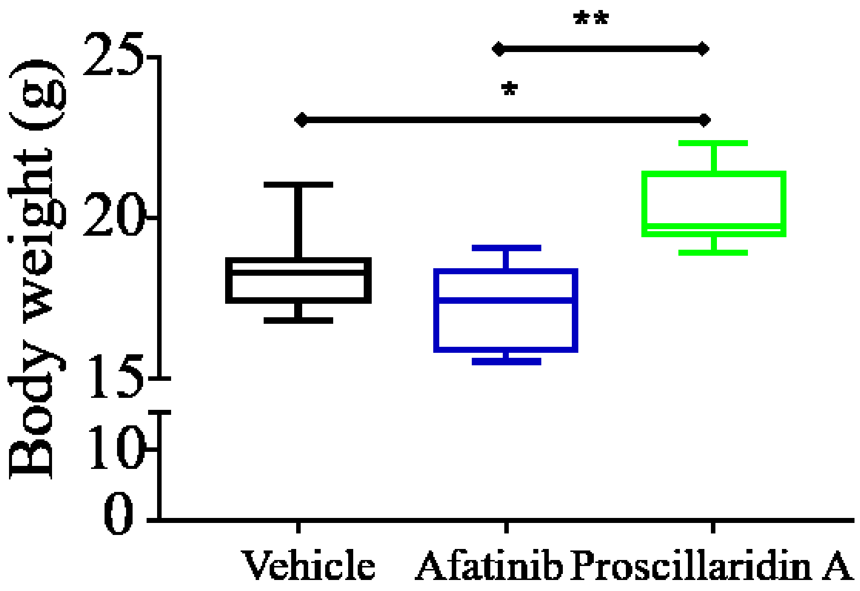

Supplementary Figure 9. Statistical analysis of the body weight of the mouse during the P.A. treatment course.

Original uncropped images were obtained from LI-COR Odyssey.

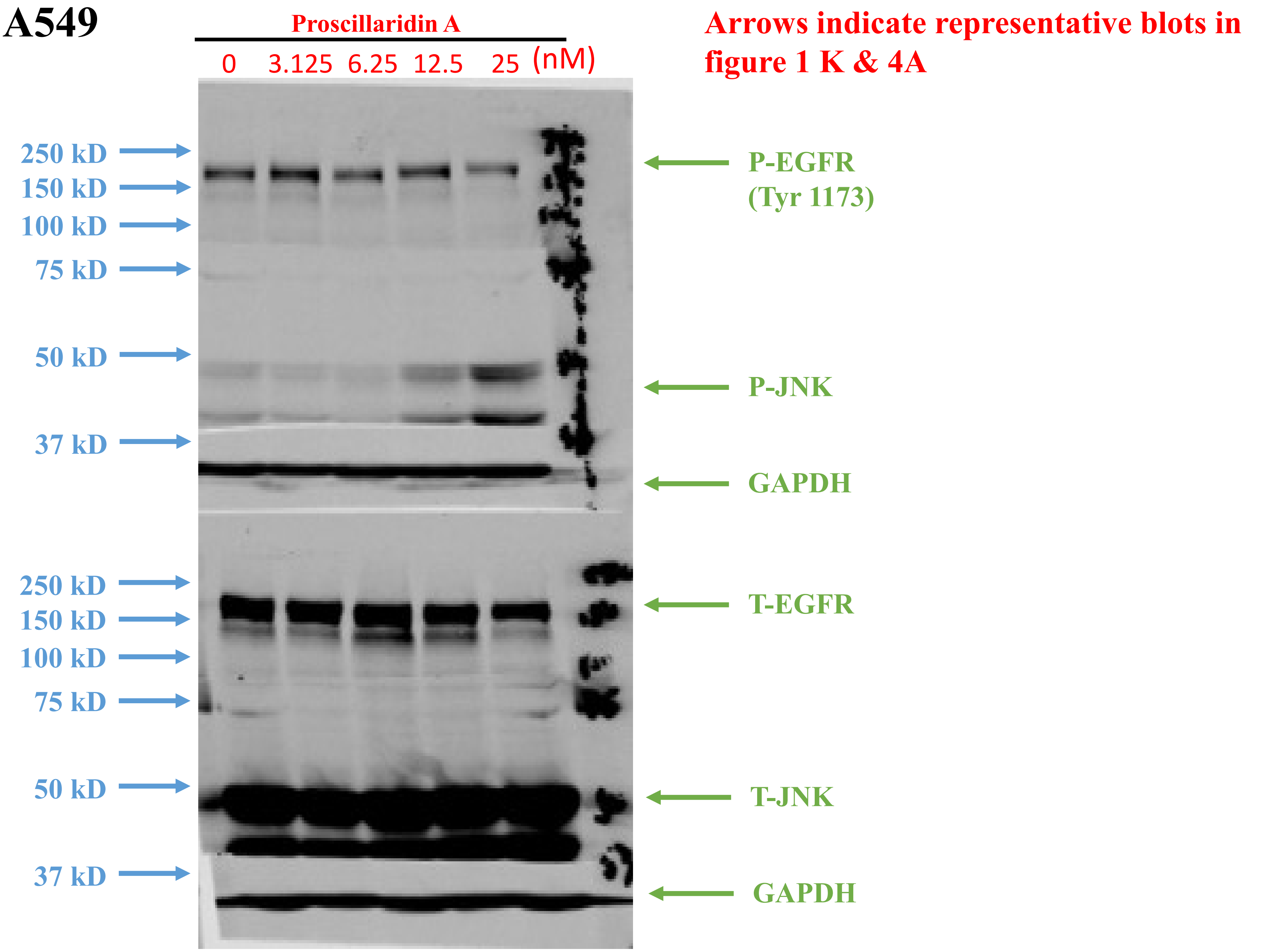
A549
Proscillaridin A
0
3.125
6.25
12.5
25
(nM)
Arrows indicate representative blots in figure 1 K & 4A
250 kD
150 kD
100 kD
75 kD
50 kD
37 kD
P-EGFR (Tyr 1173)
P-JNK
GAPDH
250 kD
150 kD
100 kD
75 kD
50 kD
37 kD
T-EGFR
T-JNK
GAPDH

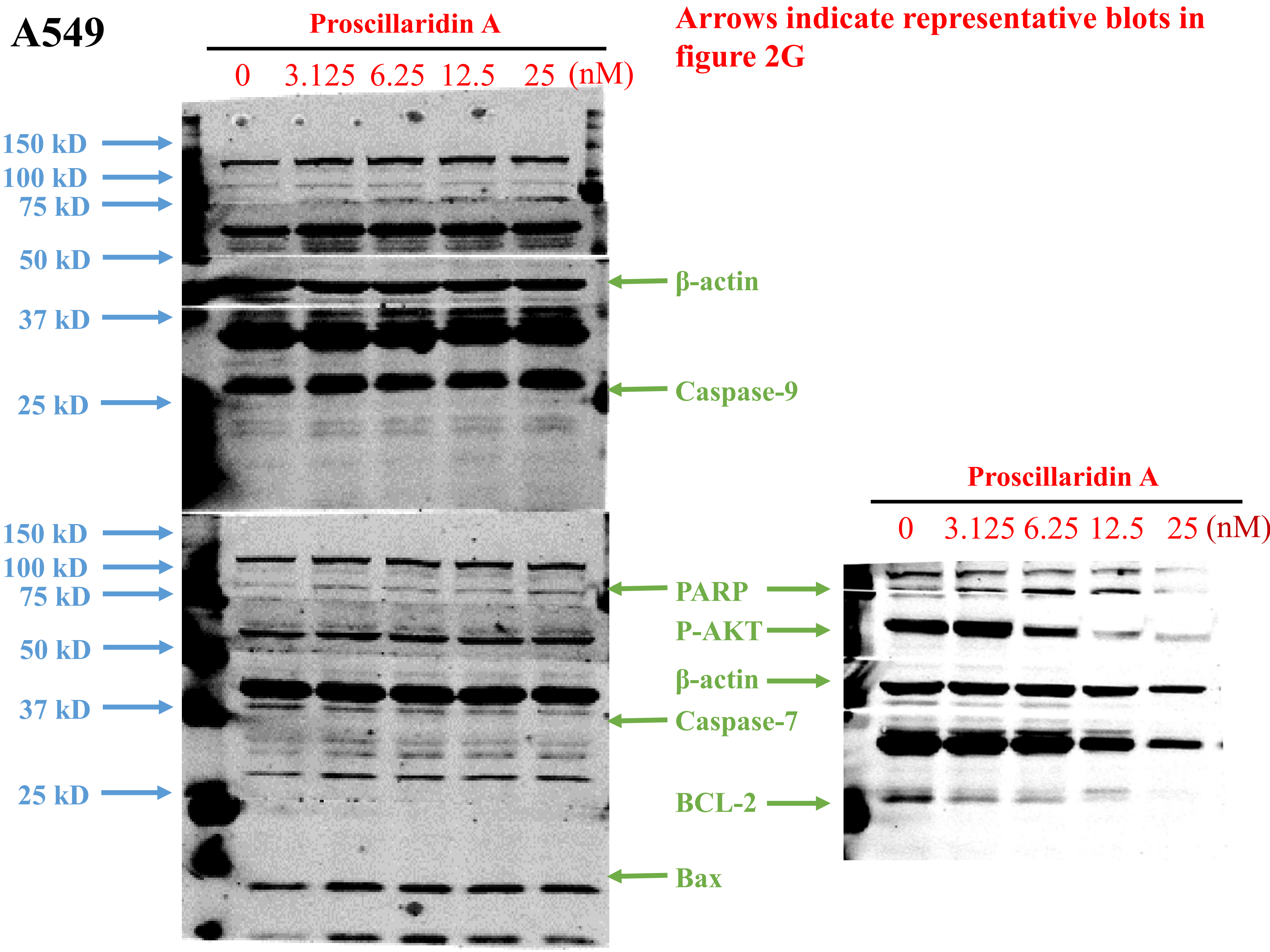

A549
Proscillaridin A
0 3.125 6.25 12.5 25 (nM)
Arrows indicate representative blots in figure 2G
150 kD
100 kD
75 kD
50 kD
37 kD
25 kD
β-actin
Caspase-9
150 kD
100 kD
75 kD
50 kD
37 kD
25 kD
PARP
P-AKT
β-actin
Caspase-7
BCL-2
Bax
Proscillaridin A
0 3.125 6.25 12.5 25 (nM)

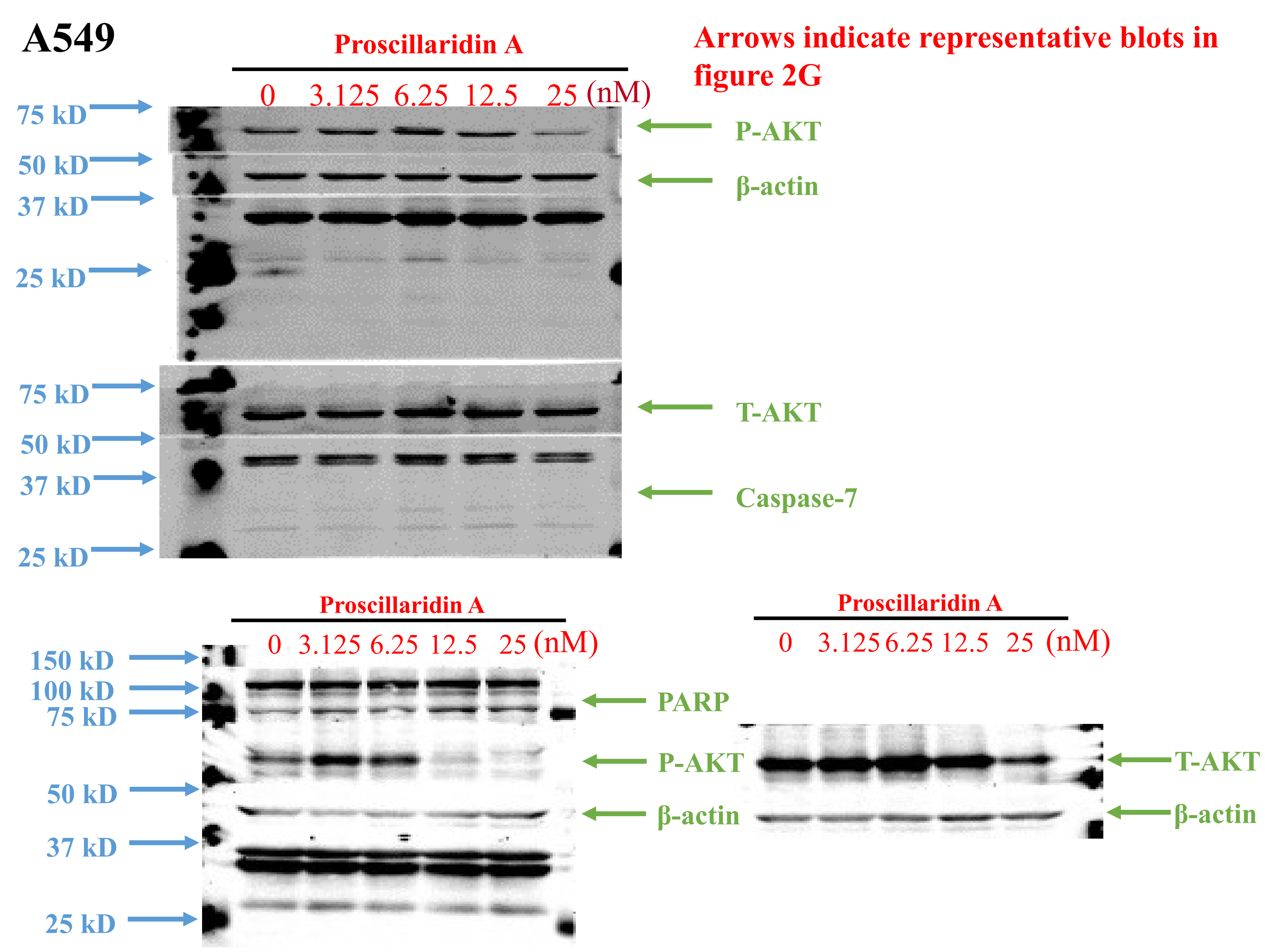
A549
Proscillaridin A
0 3.125 6.25 12.5 25 (nM)
Arrows indicate representative blots in figure 2G
75 kD
50 kD
37 kD
25 kD
P-AKT
β-actin
75 kD
50 kD
37 kD
25 kD
T-AKT
Caspase-7
Proscillaridin A
0 3.125 6.25 12.5 25 (nM)
150 kD
100 kD
75 kD
50 kD
37 kD
25 kD
PARP
P-AKT
β-actin
Proscillaridin A
0 3.125 6.25 12.5 25 (nM)
T-AKT
β-actin

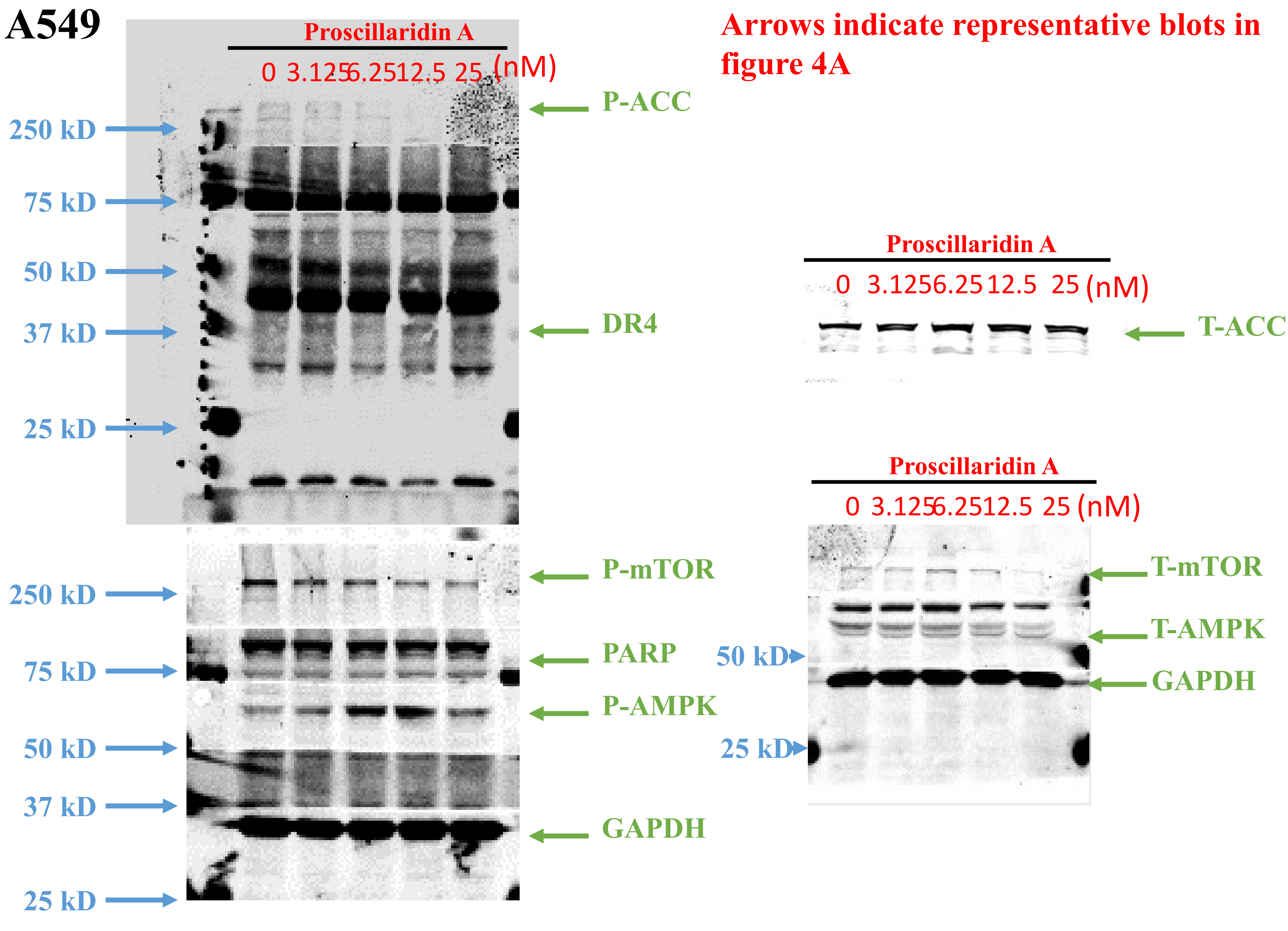
A549
Arrows indicate representative blots in figure 4A
Proscillaridin A
0 3.125 6.25 12.5 25 (nM)
250 kD
75 kD
50 kD
37 kD
25 kD
P-ACC
DR4
P-mTOR
PARP
P-AMPK
GAPDH
Proscillaridin A
0 3.125 6.25 12.5 25 (nM)
T-ACC
Proscillaridin A
0 3.125 6.25 12.5 25 (nM)
T-mTOR
T-AMPK
GAPDH
50 kD
25 kD

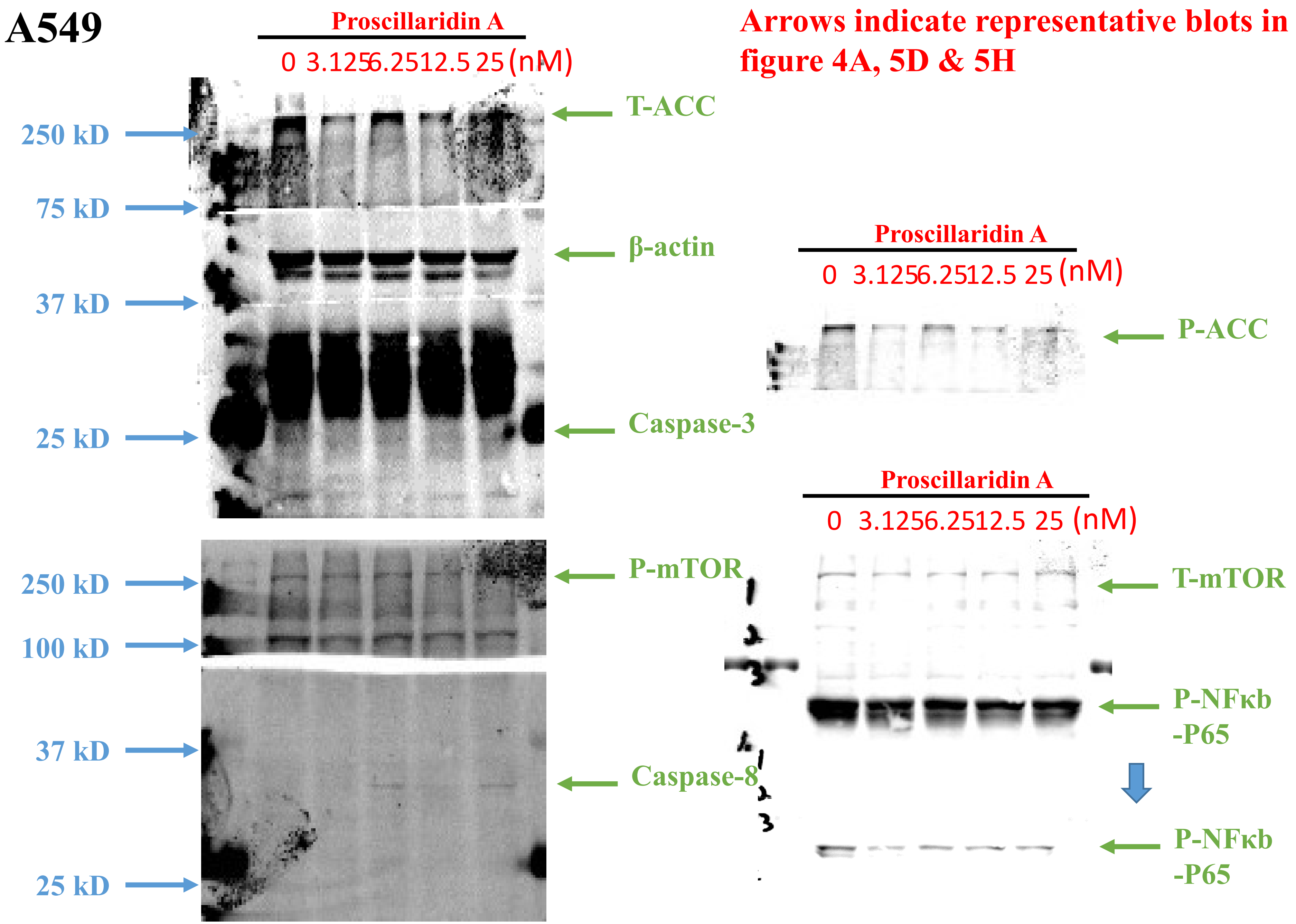

A549
Proscillaridin A
0 3.125 6.25 12.5 25 (nM)
Arrows indicate representative blots in figure 4A, 5D & 5H
250 kD
75 kD
37 kD
25 kD
T-ACC
β-actin
Caspase-3
250 kD
100 kD
37 kD
25 kD
P-mTOR
Caspase-8
Proscillaridin A
0 3.125 6.25 12.5 25 (nM)
P-ACC
Proscillaridin A
0 3.125 6.25 12.5 25 (nM)
T-mTOR
P-NFκb -P65
P-NFκb -P65

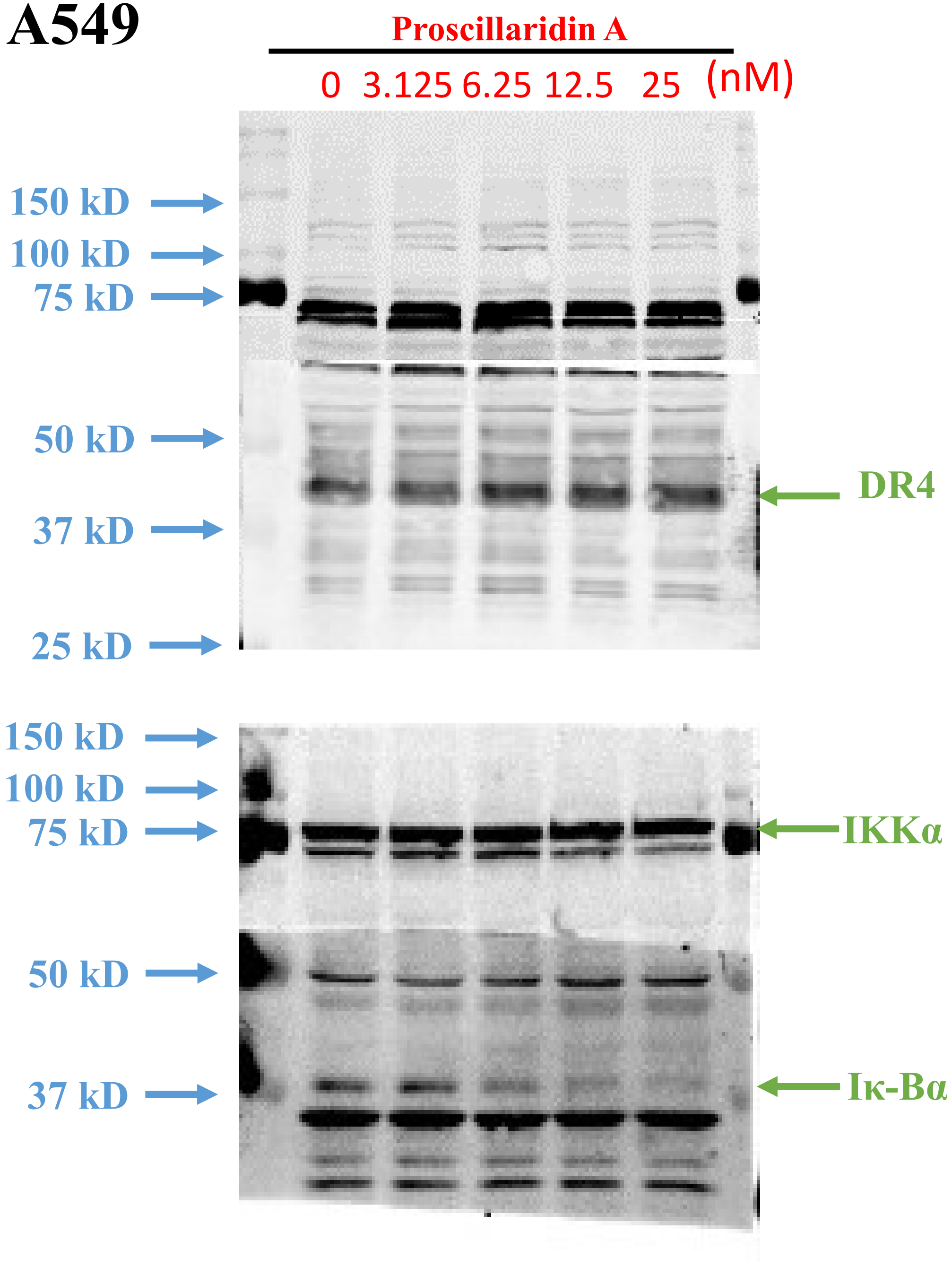

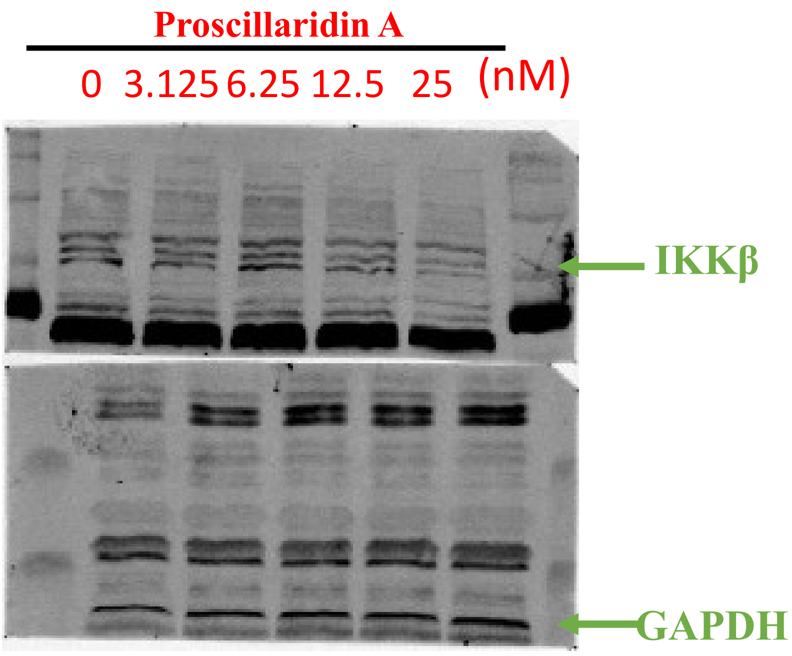

Arrows indicate representative blots in figure 5D & 5H

H1975

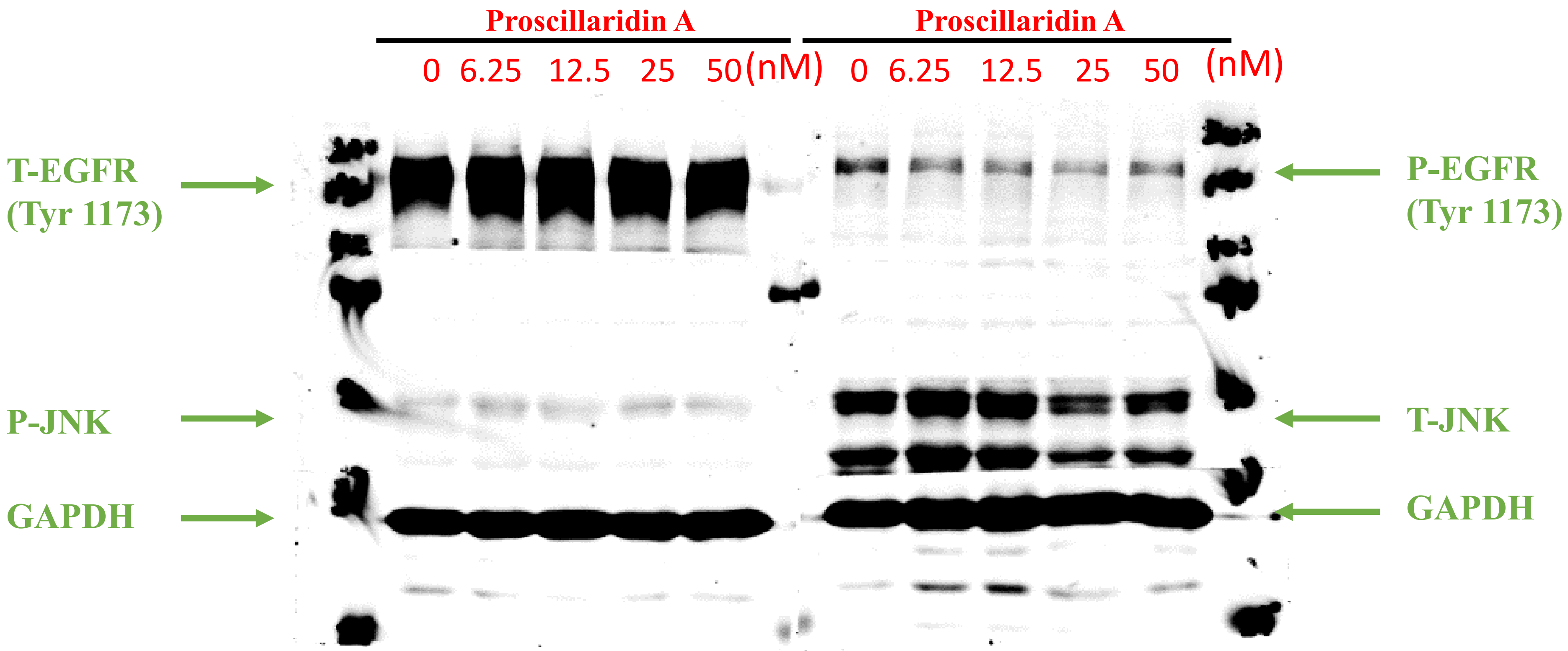

Arrows indicate representative blots in figure 1 I

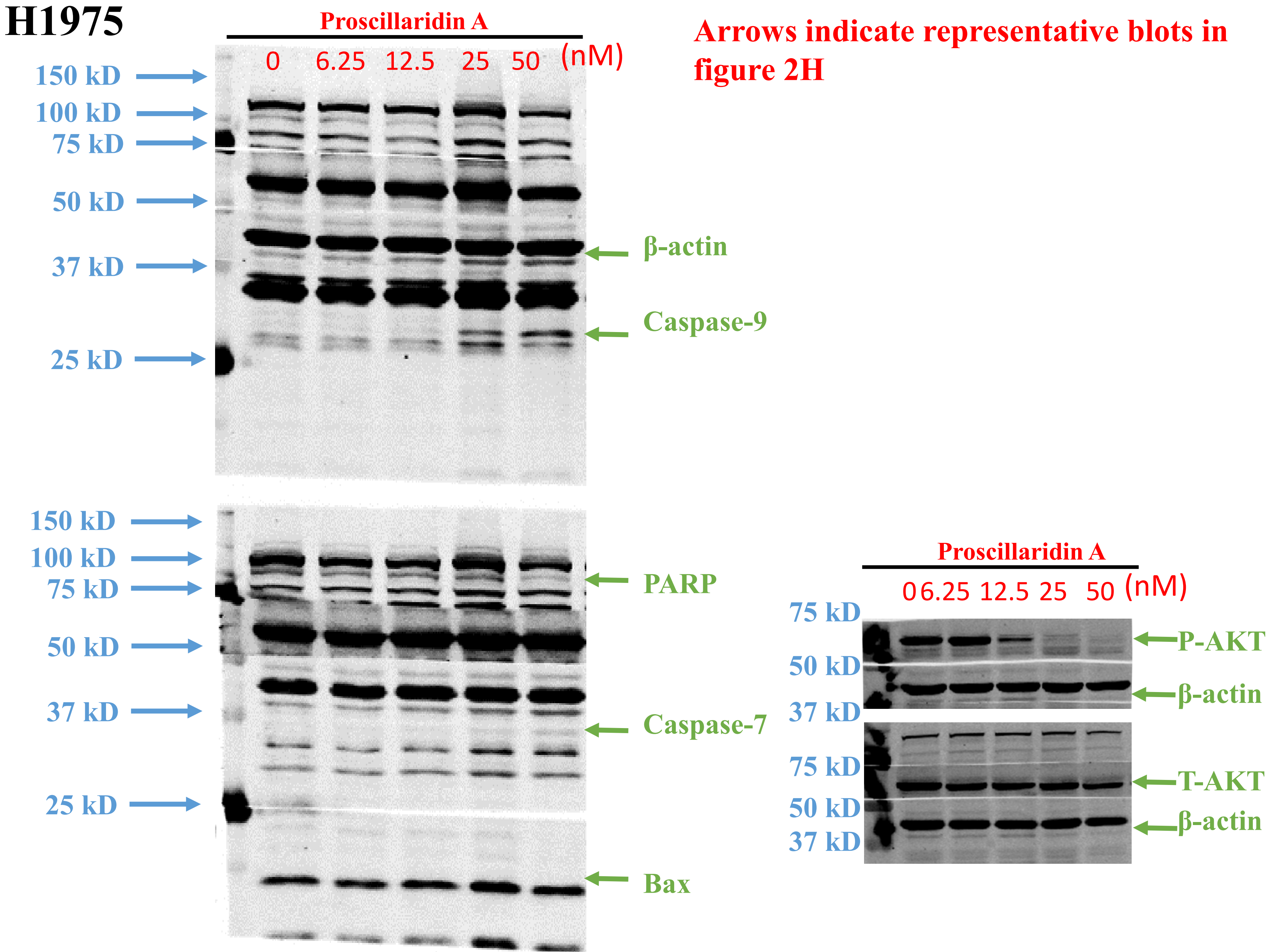
H1975
Proscillaridin A
0
6.25
12.5
25
50
(nM)
Arrows indicate representative blots in figure 2H
150 kD
100 kD
75 kD
50 kD
37 kD
25 kD
β-actin
Caspase-9
150 kD
100 kD
75 kD
50 kD
37 kD
25 kD
PARP
Caspase-7
Bax
Proscillaridin A
0
6.25
12.5
25
50
(nM)
75 kD
50 kD
37 kD
P-AKT
β-actin
75 kD
50 kD
37 kD
T-AKT
β-actin

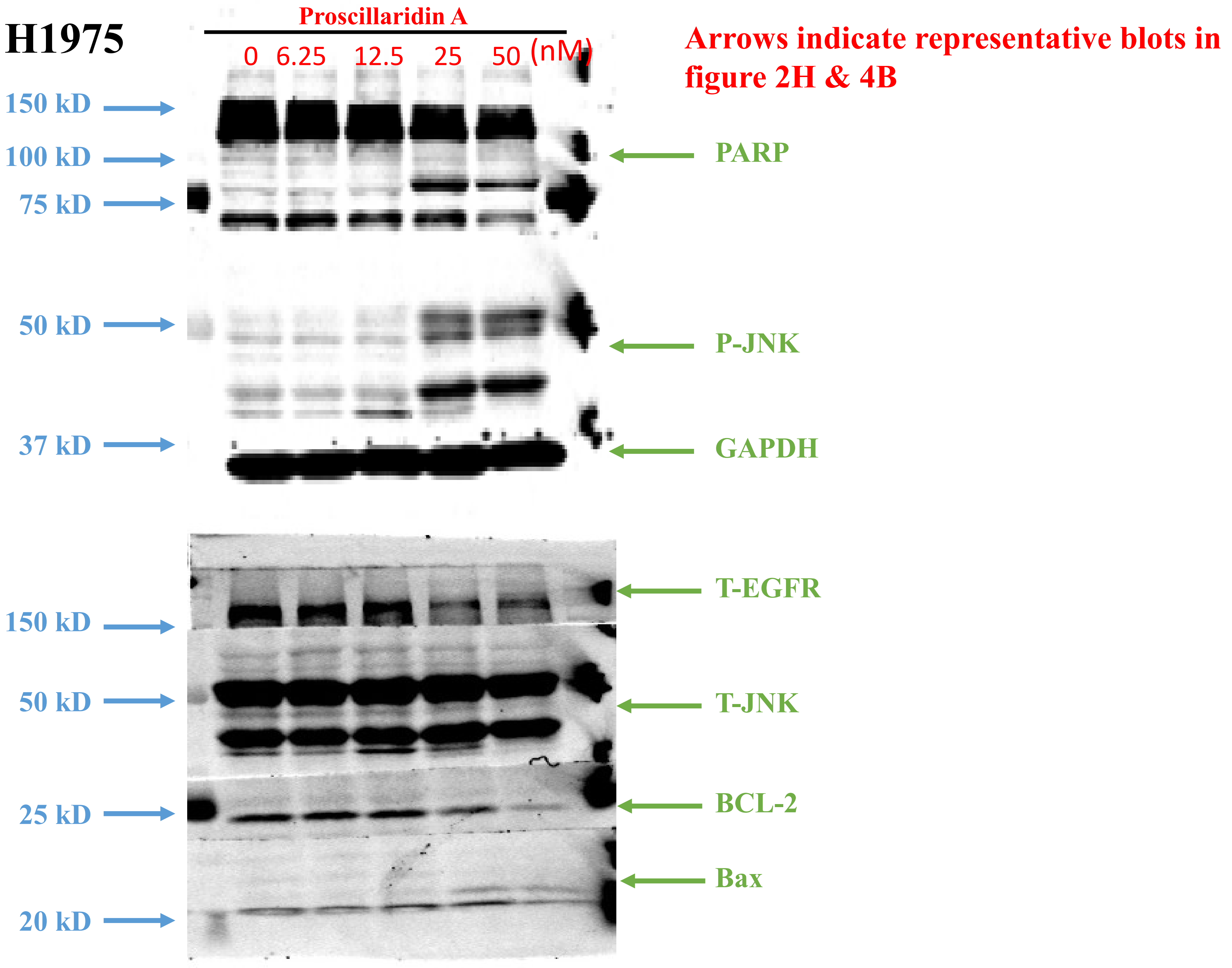

H1975
Proscillaridin A
0
6.25
12.5
25
50 (nM)
Arrows indicate representative blots in figure 2H & 4B
150 kD
100 kD
75 kD
50 kD
37 kD
PARP
P-JNK
GAPDH
150 kD
50 kD
25 kD
20 kD
T-EGFR
T-JNK
BCL-2
Bax

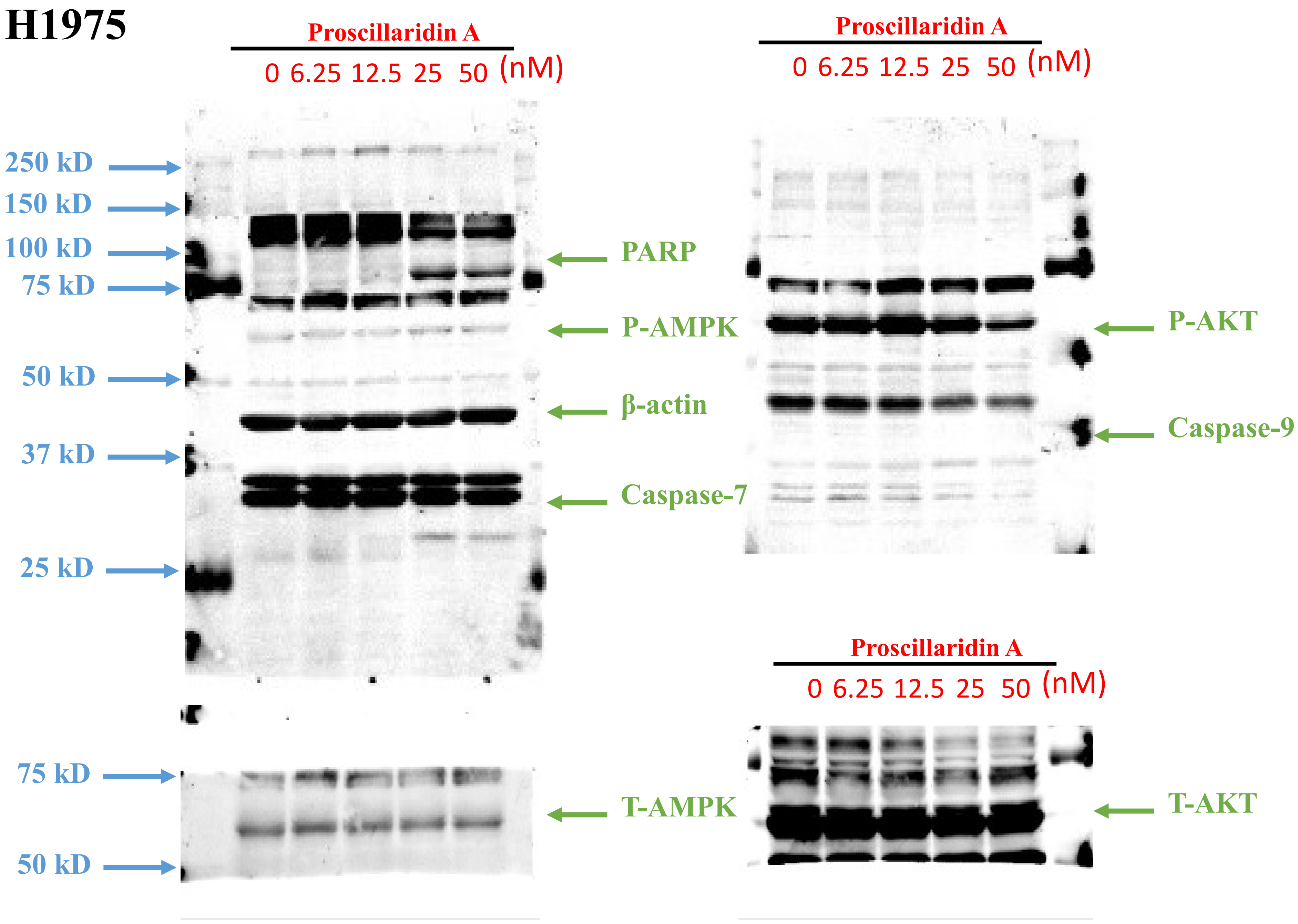

Arrows indicate representative blots in figure 4B

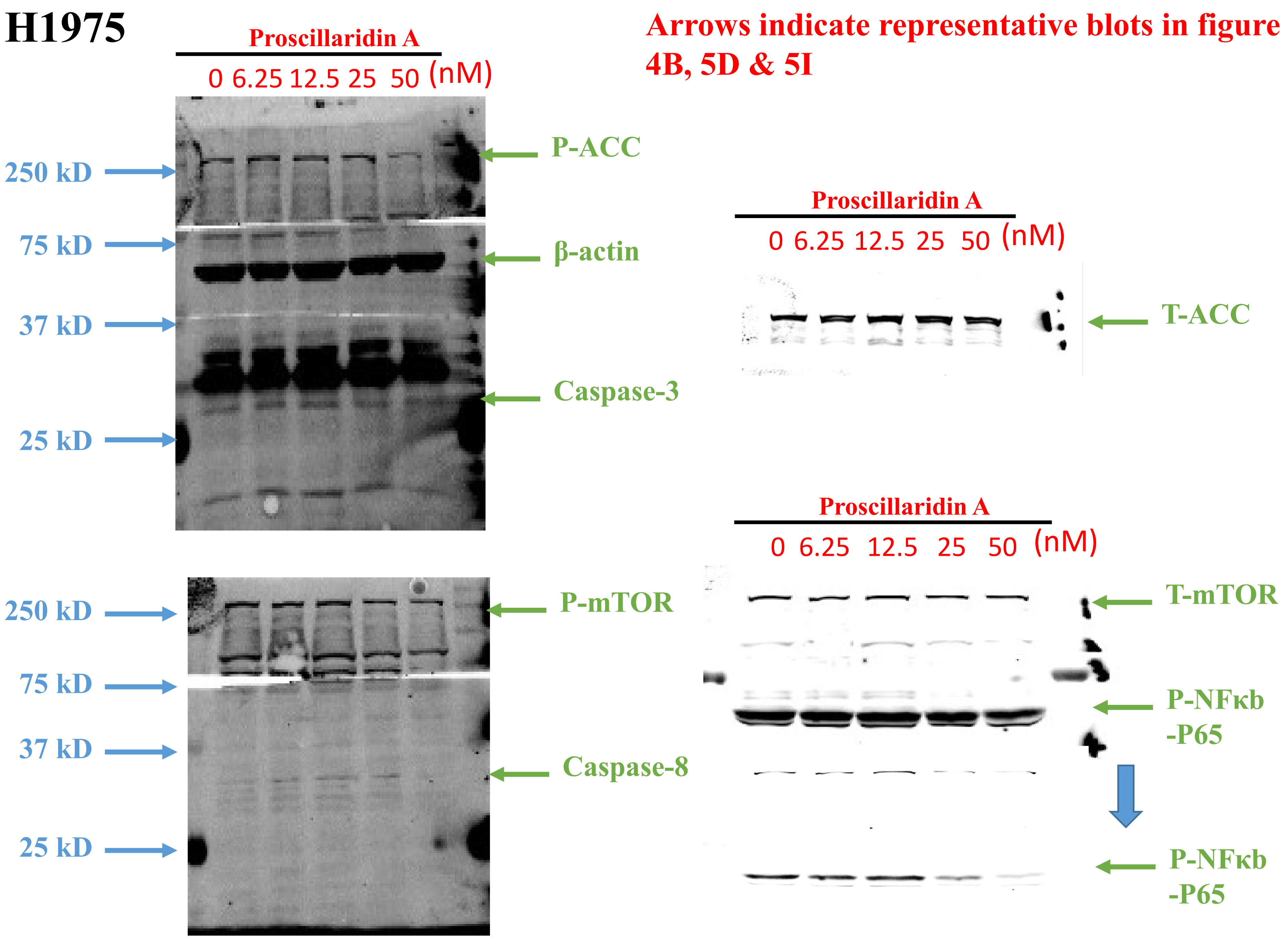
H1975
Arrows indicate representative blots in figure 4B, 5D & 5I
Proscillaridin A
0 6.25 12.5 25 50 (nM)
250 kD
75 kD
37 kD
25 kD
P-ACC
β-actin
Caspase-3
250 kD
75 kD
37 kD
25 kD
P-mTOR
Caspase-8
Proscillaridin A
0 6.25 12.5 25 50 (nM)
T-ACC
Proscillaridin A
0 6.25 12.5 25 50 (nM)
T-mTOR
P-NFκb -P65
P-NFκb -P65

H1975

Arrows indicate representative blots in figure 5D & 5I

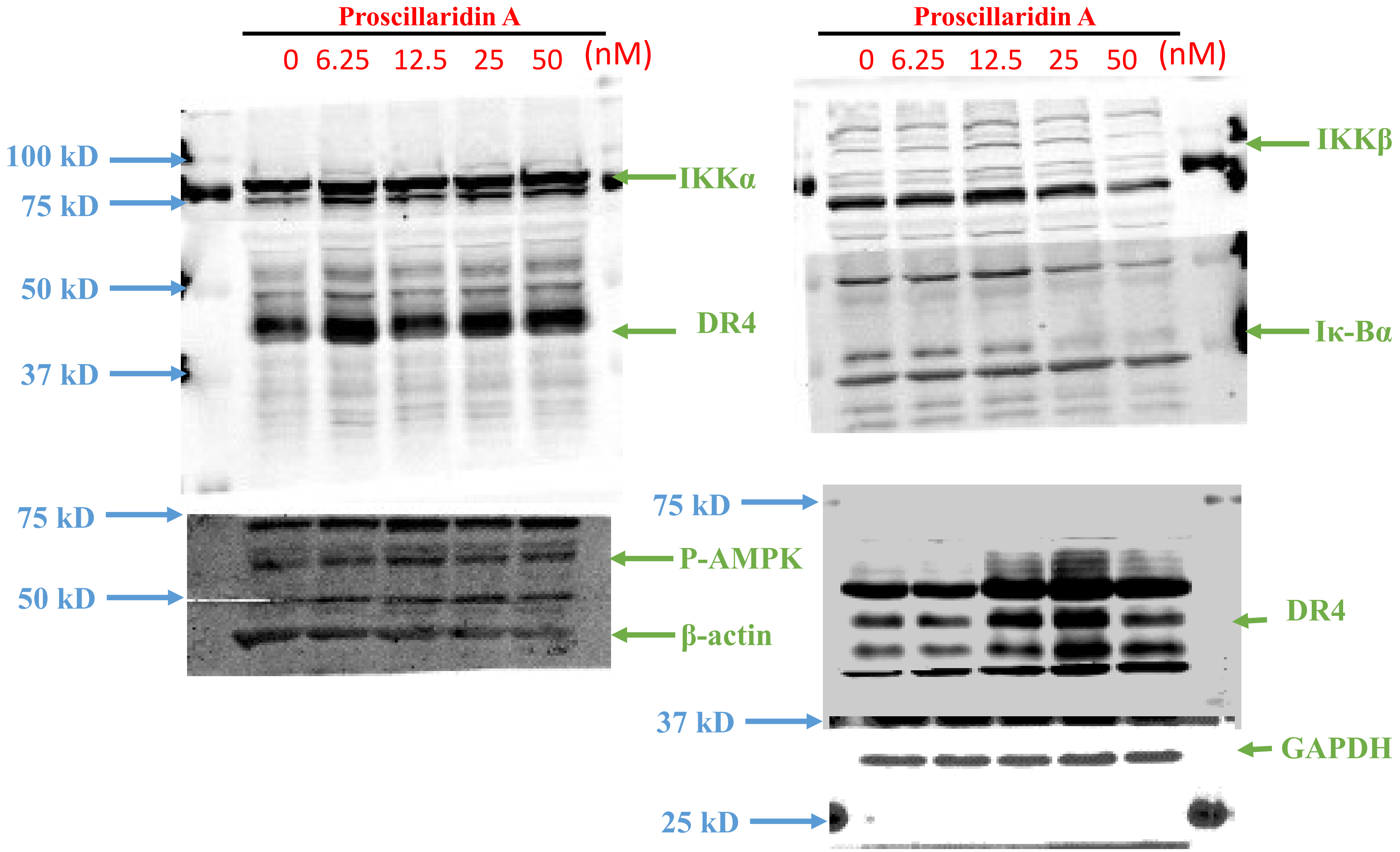

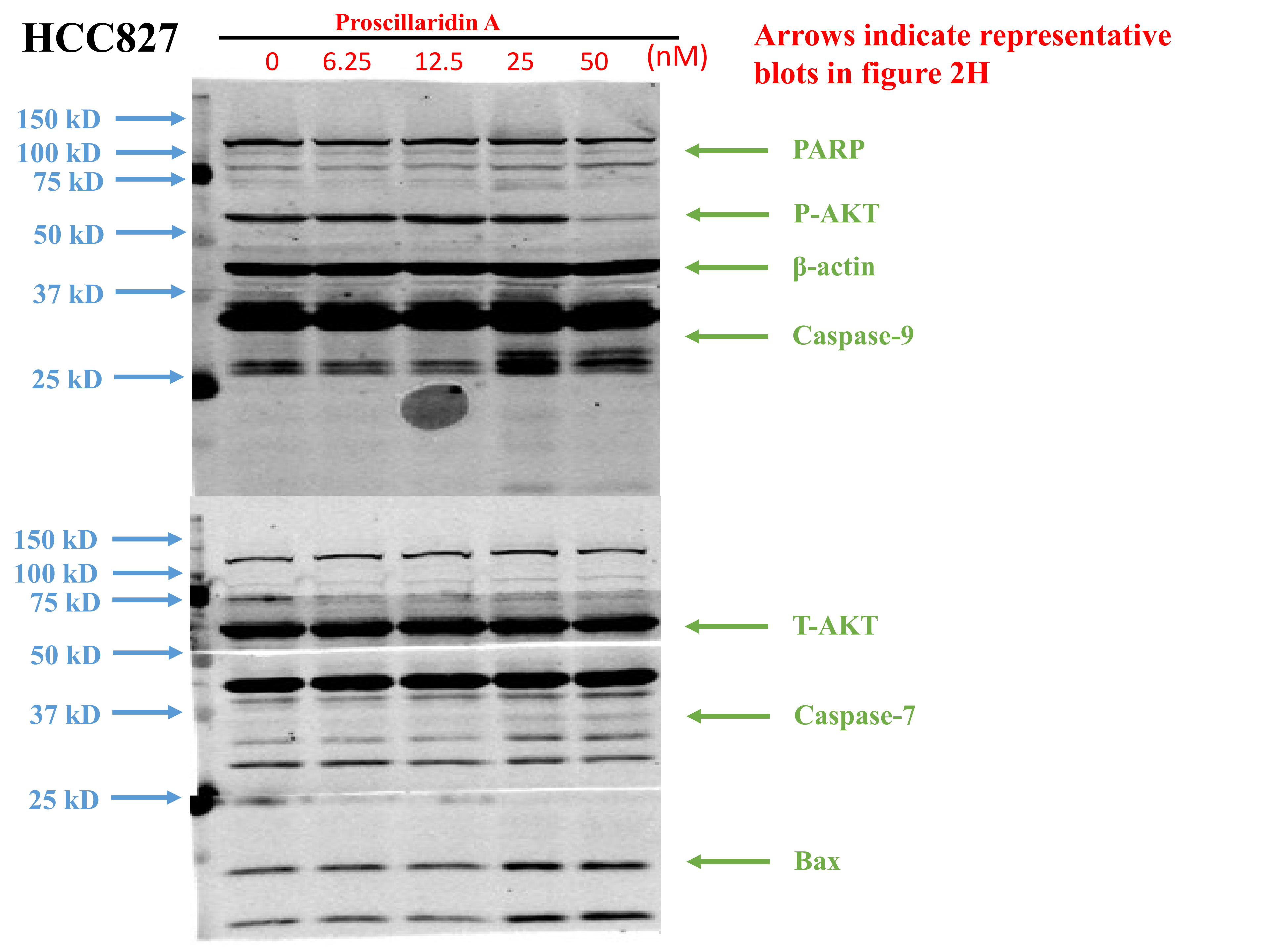
HCC827
Proscillaridin A
0
6.25
12.5
25
50
(nM)
Arrows indicate representative blots in figure 2H
150 kD
100 kD
75 kD
50 kD
37 kD
25 kD
PARP
P-AKT
β-actin
Caspase-9
150 kD
100 kD
75 kD
50 kD
37 kD
25 kD
T-AKT
Caspase-7
Bax

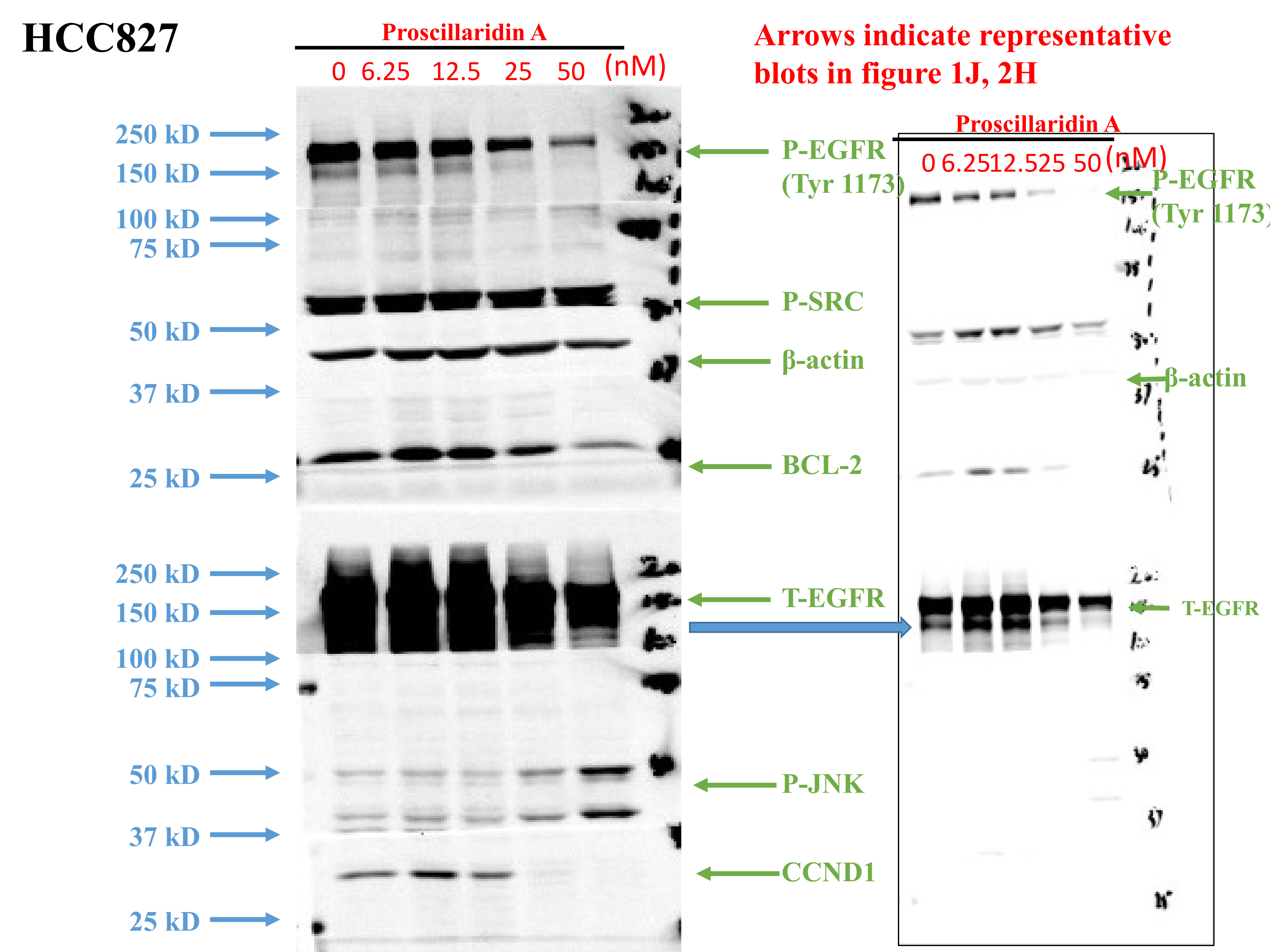
HCC827
Proscillaridin A
0 6.25 12.5 25 50 (nM)
Arrows indicate representative blots in figure 1J, 2H
250 kD
150 kD
100 kD
75 kD
50 kD
37 kD
25 kD
P-EGFR (Tyr 1173)
P-SRC
β-actin
BCL-2
T-EGFR
P-JNK
CCND1
Proscillaridin A
0 6.25 12.5 25 50 (nM)
P-EGFR (Tyr 1173)
β-actin
T-EGFR

In vivo

Arrows indicate representative blots in figure 6 F

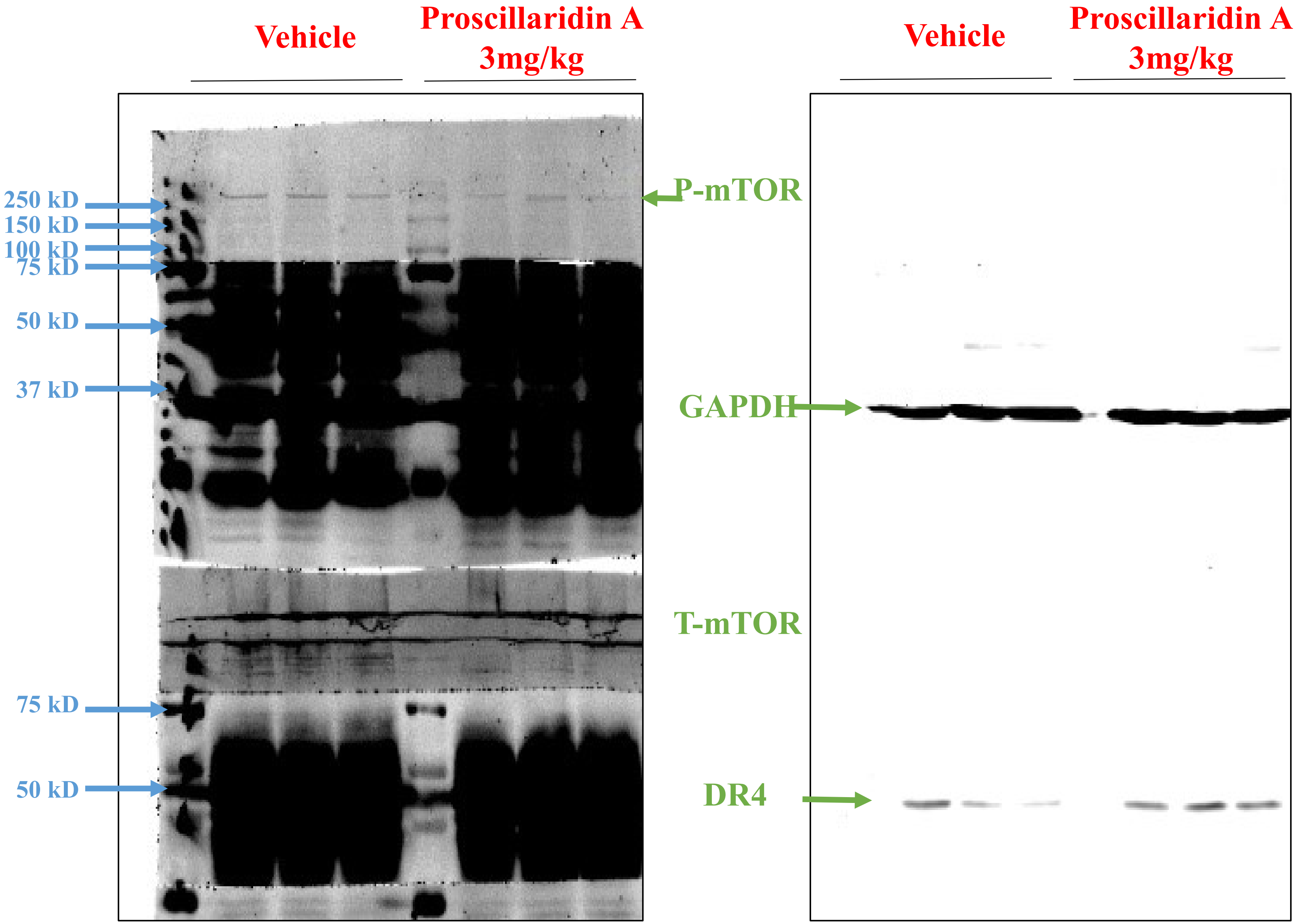

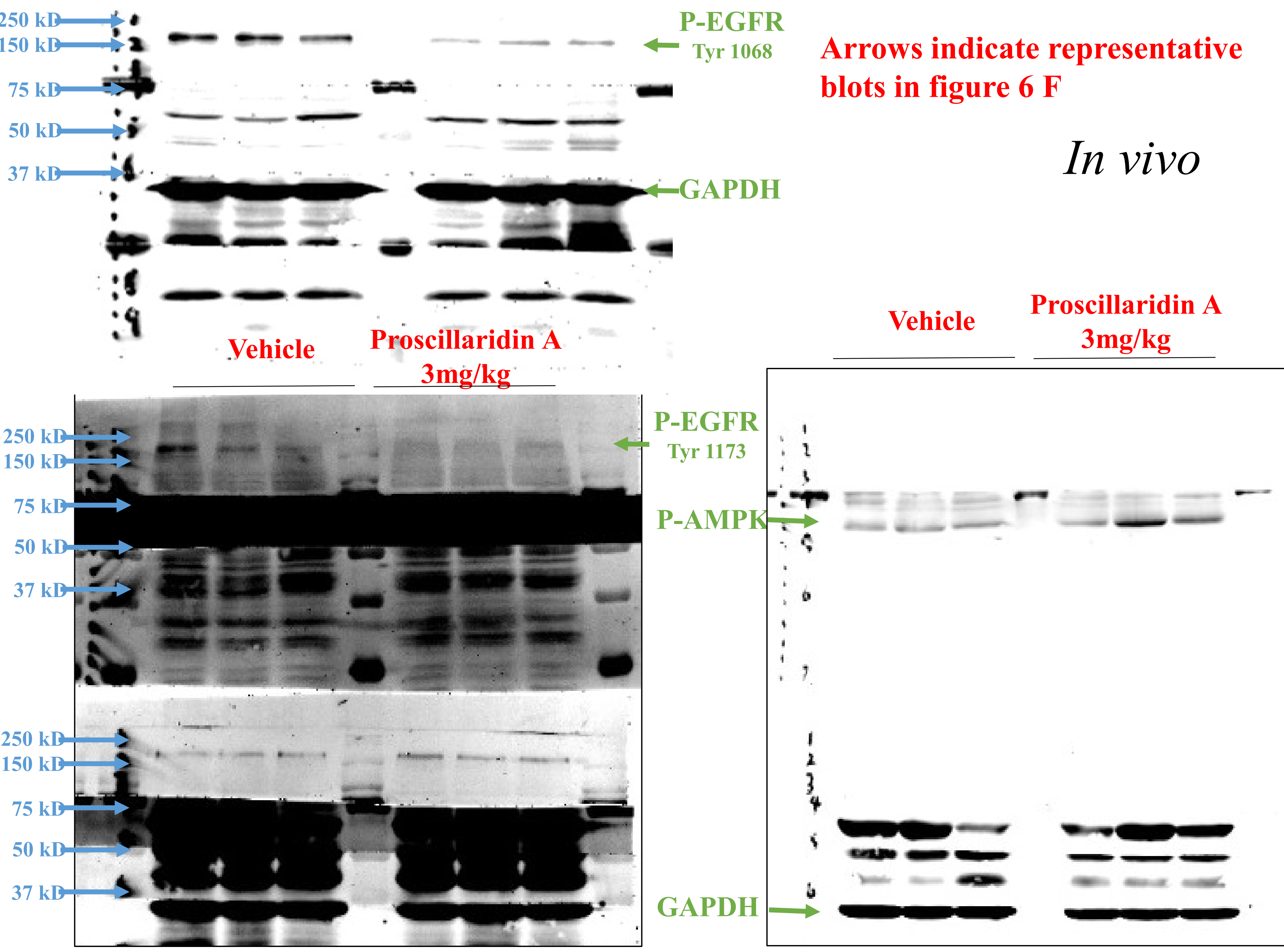

Arrows indicate representative blots in figure 6 F
In vivo
250 kD
150 kD
75 kD
50 kD
37 kD
P-EGFR
Tyr 1068
GAPDH
Vehicle
Proscillaridin A
3mg/kg
250 kD
150 kD
75 kD
50 kD
37 kD
P-EGFR
Tyr 1173
250 kD
150 kD
75 kD
50 kD
37 kD
Vehicle
Proscillaridin A
3mg/kg
P-AMPK
GAPDH

In vivo

Arrows indicate representative blots in figure 6 F

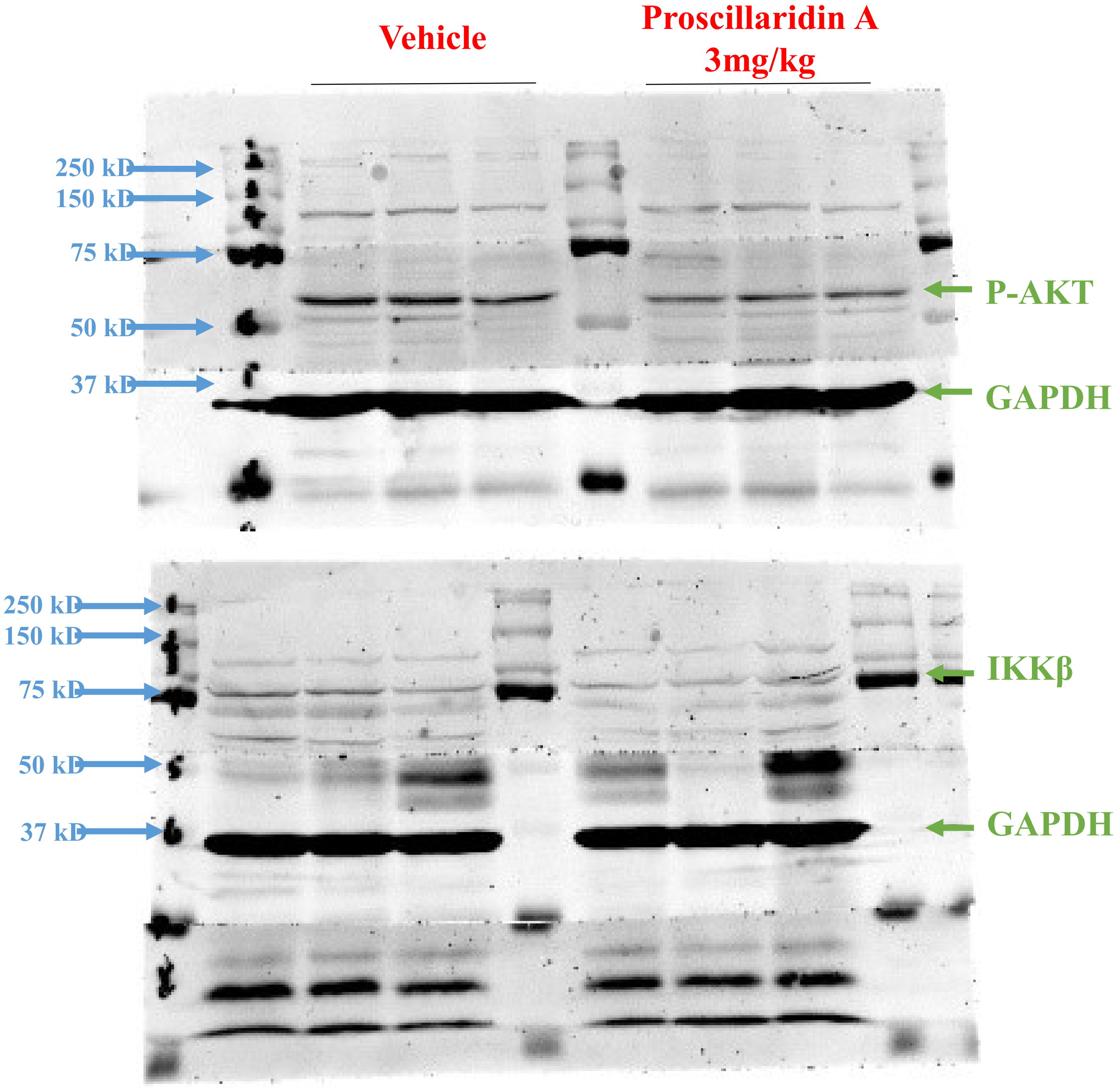

In vivo

Arrows indicate representative blots in figure 6 F

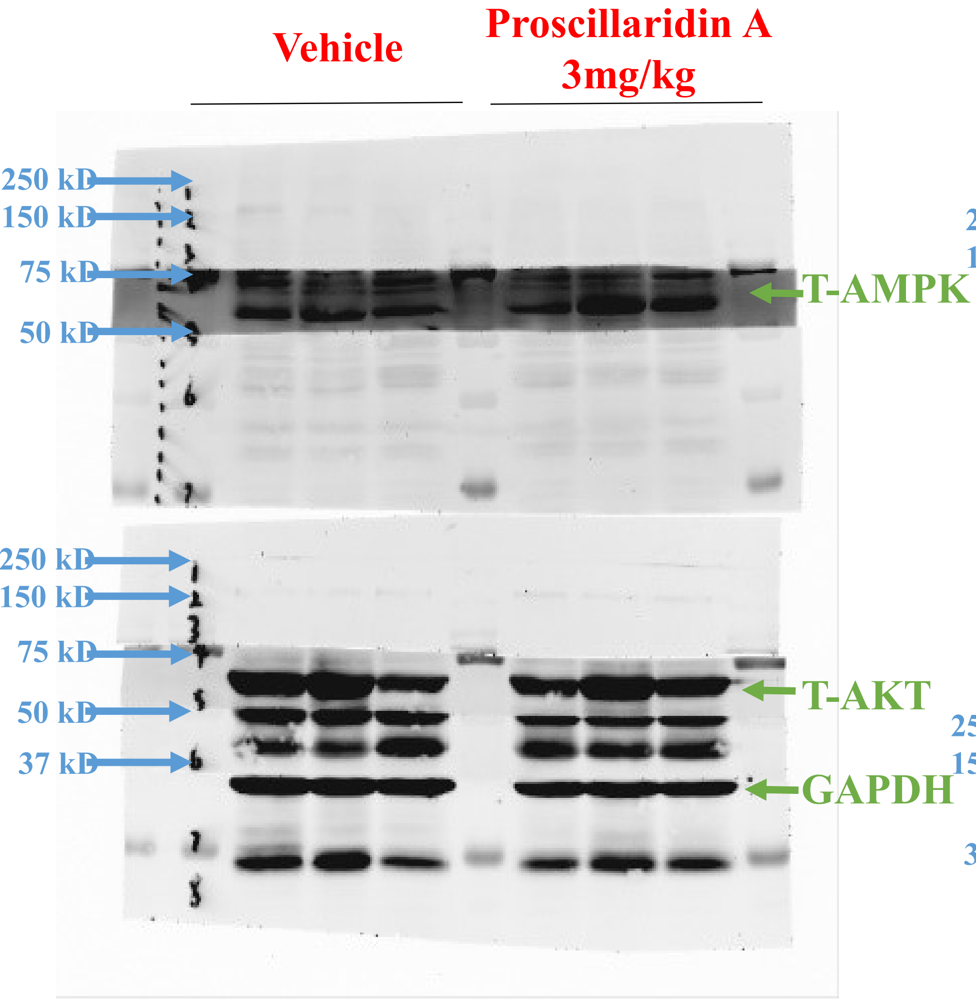

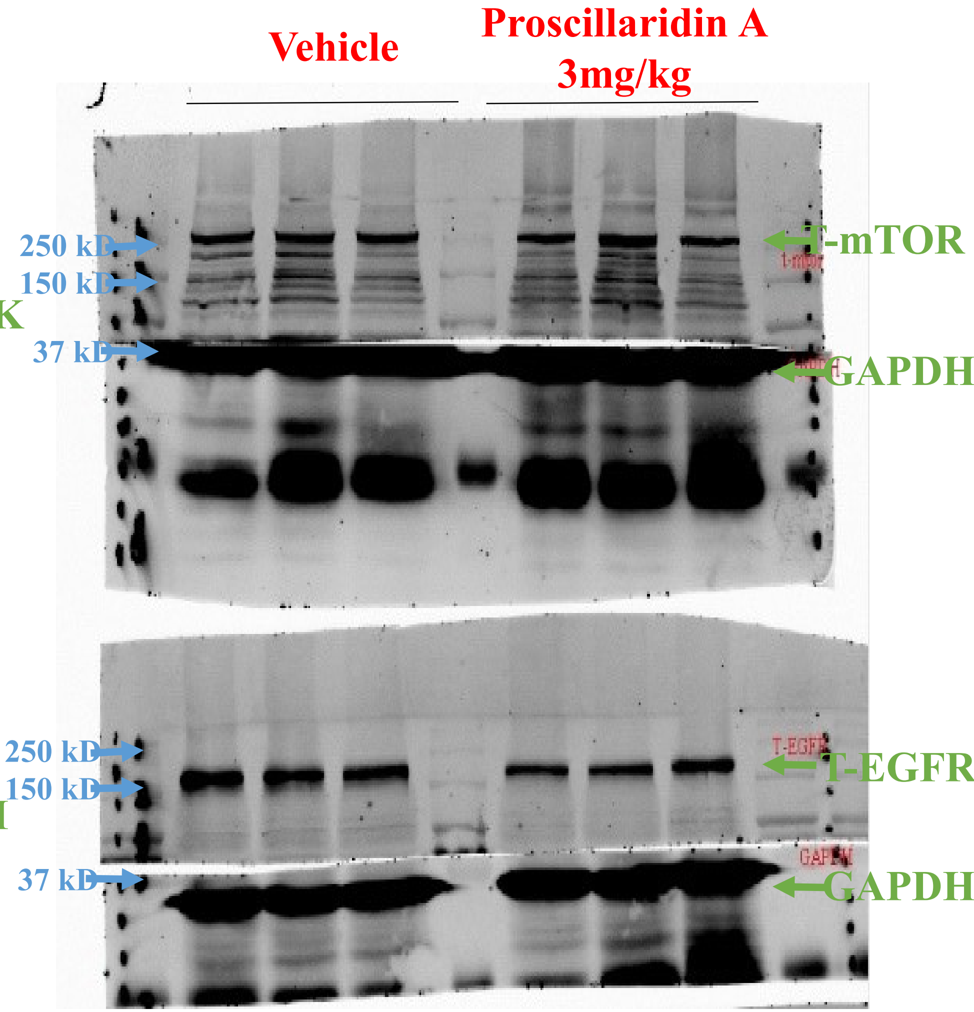

Supplement: Supplementary file 1 — Supplementary material [file 41419_2018_733_MOESM1_ESM.pdf]
