# Supplementary material for: Proscillaridin A induces apoptosis and suppresses non-small-cell lung cancer tumor growth via calcium-induced DR4 upregulation
Source: Cell Death Dis. 2018 Jun 13;9(6):696. doi: 10.1038/s41419-018-0733-4 (PMC5999972; doi:10.1038/s41419-018-0733-4)
Supplement: Supplementary file 2 — Supplemental figure legends [file 41419_2018_733_MOESM2_ESM.docx]

**Supplementary table & figure legends:**

**Supplementary Table 1:** The CC_50_ value of P.A on NSCLC cell lines and CCD19-LU normal lung fibroblast cell line.

**Supplementary Figure 1:** The treatment effect of P.A at different time points in A549 cell line.

**Supplementary Figure 2:** The treatment effect of P.A at different time points in H1975 cell line.

**Supplementary Figure 3:** The flow cytometry analysis of the cytotoxicity effect of BM, Compound C and JNK inhibitor.

**Supplementary Figure 4:** The effect of DR4 siRNA knockdown.

**Supplementary Figure 5:** The localization of BAX after the treatment of P.A.

**Supplementary Figure 6:** The effect of P.A on the unfolded protein response.

**Supplementary Figure 7:** The combination treatment effect of P.A and DR4 ligands in A549 cell line.

**Supplementary Figure 8:** The combination treatment effect of P.A and DR4 ligands in HCC827 cell line.

**Supplementary Figure 9:** Statistical analysis of the body weight of the mouse during the P.A. treatment course.
